# Rapid identification of intact bacterial resistance plasmids via optical mapping of single DNA molecules

**DOI:** 10.1038/srep30410

**Published:** 2016-07-27

**Authors:** Lena K. Nyberg, Saair Quaderi, Gustav Emilsson, Nahid Karami, Erik Lagerstedt, Vilhelm Müller, Charleston Noble, Susanna Hammarberg, Adam N. Nilsson, Fei Sjöberg, Joachim Fritzsche, Erik Kristiansson, Linus Sandegren, Tobias Ambjörnsson, Fredrik Westerlund

**Affiliations:** 1 Department of Biology and Biological Engineering, Chalmers University of Technology, Gothenburg, Sweden; 2 Department of Astronomy and Theoretical Physics, Lund University, Lund, Sweden; 3 Department of Applied Physics, Chalmers University of Technology, Gothenburg, Sweden; 4 Department of Infectious Diseases, Sahlgrenska Academy, University of Gothenburg, Gothenburg, Sweden; 5 Department of Mathematical Sciences, Chalmers University of Technology/University of Gothenburg, Gothenburg, Sweden; 6 Department of Medical Biochemistry and Microbiology, Uppsala University, Uppsala, Sweden

## Abstract

The rapid spread of antibiotic resistance – currently one of the greatest threats to human health according to WHO – is to a large extent enabled by plasmid-mediated horizontal transfer of resistance genes. Rapid identification and characterization of plasmids is thus important both for individual clinical outcomes and for epidemiological monitoring of antibiotic resistance. Toward this aim, we have developed an optical DNA mapping procedure where individual intact plasmids are elongated within nanofluidic channels and visualized through fluorescence microscopy, yielding *barcodes* that reflect the underlying sequence. The assay rapidly identifies plasmids through statistical comparisons with barcodes based on publicly available sequence repositories and also enables detection of structural variations. Since the assay yields holistic sequence information for individual *intact* plasmids, it is an ideal complement to next generation sequencing efforts which involve reassembly of sequence reads from fragmented DNA molecules. The assay should be applicable in microbiology labs around the world in applications ranging from fundamental plasmid biology to clinical epidemiology and diagnostics.

The World Health Organization (WHO) has classified antibiotic resistance as one of the greatest global threats to human health by warning that a “post-antibiotic era”, where common infections and minor injuries can kill, may soon be a reality^1^,^2^. One main reason for the rapid increase of antibiotic resistant bacteria is the presence of resistance genes located on mobile genetic elements, such as plasmids^3^. Plasmids are circular DNA molecules, 1–1000 kilobasepairs (kbp) in size, that are actively and efficiently transferred between bacterial cells, of the same or different genera, through the process of conjugation^4^. Plasmid-borne resistance, especially among Gram-negative bacteria, has rapidly increased towards the majority of clinically available treatments such as sulphonamides, trimethoprim, fluoroquinolones, aminoglycosides, chloramphenicol, macrolides, tetracyclines, penicillins, cephalosporins and carbapenems^5^,^6^. Alarmingly, Liu *et al.* recently reported the first example of a plasmid-borne resistance determinant to polymyxins, the very last group of antibiotics available for treating carbapenem resistant bacteria^7^. This poses a severe threat since pan-resistant bacteria may now form by combining the highly transmissible polymyxin resistance plasmid with plasmids encoding carbapenemases.

The lack of rapid and comprehensive methods for plasmid identification and characterization is a major obstacle for epidemiological tracing of plasmid-mediated resistance^8^,^9^,^10^. Pulsed Field Gel Electrophoresis (PFGE) coupled with S1 digestion^11^ can reveal the number and sizes of plasmids in resistant bacteria, but the assay takes several days from sample collection to result and no sequence information is obtained and data from different labs are not easily compared. Recently, large-scale sequencing approaches have been introduced, but even though sequencing is becoming cheaper and less time consuming, the end result still requires extensive downstream data analysis, where short reads are assembled, that is complicated by the dynamic nature of plasmids^12^. Improved methods for faster identification and characterization of bacterial plasmids are therefore needed.

Optical DNA mapping methods are based on visualizing the coarse-grained sequence of *single* DNA molecules, stretched either on a surface^13^ or in nanofluidic channels^14^,^15^, using fluorescence microscopy. The use of optical mapping in genetics has grown rapidly in recent years^16^,^17^,^18^,^19^,^20^. The main benefit is the extreme read lengths compared to existing sequencing techniques, realized by visualizing large DNA molecules (100 kbp or larger) using sequence specific tags. We have recently introduced a simple optical DNA mapping protocol that relies on non-covalent competitive binding of the fluorescent dye YOYO-1 (YOYO) and the non-fluorescent netropsin to DNA^21^,^22^. Netropsin has a strong preference for AT-rich sequences^23^ and thereby partially blocks such sequences from YOYO binding. The resulting variation in fluorescence emission along the DNA molecules is a *DNA barcode*, where AT-rich regions are dark and GC-rich regions are bright. In contrast to most other mapping techniques, no enzymatic reactions are involved; the barcodes are formed via a simple incubation step with the dyes.

When intact plasmids are confined in nanofluidic channels, they contain twice the amount of DNA per length unit as linear DNA due to their circular structure. As a result, they are approximately twice as bright under fluorescence microscopes^24^. Therefore, remaining chromosomal DNA and plasmid fragments can be automatically discarded from the analysis of *intact* plasmids^25^.

In this study we use optical mapping to characterize plasmid DNA using the workflow schematically described in Fig. 1. We demonstrate experimentally that we are able to identify previously sequenced plasmids that are ~70 kbp or larger from a theoretical barcode database based on all sequenced plasmids. We confirm our observations by comparing all pairs of sequenced plasmids *in silico*. We also demonstrate that the assay can be used to identify structural variations that are an integral part in the dynamic nature of plasmids^26^. The method has the unique ability to provide sequence information from individual *intact* plasmids, making it a very important complement to next generation sequencing techniques. The assay can be used for fundamental research on plasmids but also in clinical and epidemiological applications.

## Results

To test the applicability of the optical DNA mapping method for identifying plasmids we used three fully sequenced and well-characterized plasmids of different sizes: RP1 from *Pseudomonas aeruginosa* (60.1 kbp)^27^, R100 from *Shigella flexneri 2b* (94.3 kbp)^28^, and pUUH239.2 from *Klebsiella pneumoniae* (220.8 kbp)^29^ (details in Supplementary Table 1). All three plasmids were originally isolated from clinical samples and carry genes coding for antibiotic resistance. The plasmids were introduced into the channels in their circular form and subsequently linearized using photonicking, thus making sure that only intact plasmids were analyzed (details in Methods), to avoid analyzing linear chromosomal DNA fragments or fragmented plasmids that may remain in the sample after plasmid extraction. We directly obtained the size of each individual plasmid by measuring the extension of its linear form along the nanochannel and compared it to the extension of a reference DNA sequence of known size (see Methods)^25^. The reported sizes for each of the three types of plasmids are within one standard deviation of the sizes obtained here (Fig. 2a).

From the same experiments used for determining the sizes, we obtained the barcodes of the intact plasmids for each individual DNA molecule (Supplementary Methods 1–2)^21^,^22^. The individual barcodes were merged into *consensus* barcodes for each type of plasmid, consisting of up to 25 individual barcodes, to minimize the effect of molecule-to-molecule variations and eliminate any distortions at the ends of the individual barcodes (see Methods and Supplementary Methods 2–5). Experimental consensus barcodes for RP1, R100 and pUUH239.2 are shown in Fig. 2b–d, demonstrating an excellent overall similarity with the corresponding theoretical barcodes created from the plasmid sequence database (see Methods)^22^. While the main analysis in this paper is based on the use of consensus barcodes, we noted that the similarity between each *individual* barcode and the corresponding theoretical barcode was also very good (see Supporting Figure S1).

### Comparing experimental consensus barcodes to theoretical barcodes

To assess the similarity of an experimental and a theoretical barcode we calculated a bit-weighted best Pearson correlation coefficient (Ĉ), where a Ĉ-value of 1 corresponds to a perfect match (see Methods and Supplementary Methods 4)^22^. We compared the experimental consensus barcodes to theoretical barcodes and created histograms of all Ĉ-values obtained (Fig. 3a–c) and restricted the database comparison to a size interval of ±3 standard deviations from the experimentally determined mean size of each tested plasmid. Starting with the consensus barcode for R100, two plasmid sequences in the database stood out with a very high Ĉ-value (Fig. 3b): the reference sequences of plasmids R100 and NR1, where the latter is a variant of R100 with an overall sequence identity of 99.8%. For the larger plasmid pUUH239.2, the corresponding reference sequence also yields the highest Ĉ-value (Fig. 3c). However, compared to R100, the fit to the reference sequence gives a much lower Ĉ-value for pUUH239.2 while the average Ĉ-value for the other sequences is also lower. For the smallest plasmid, RP1 (Fig. 3a), the correct sequence ends up in the top 0.5% in Ĉ-value but does not stand out from the “crowd”, as is the case for R100 and pUUH239.2. Since we were able to identify both R100 and pUUH239.2 from their experimental consensus barcodes we were interested in how many barcodes that were necessary for identification. Interestingly, we found that a vast majority of the *individual* barcodes for these two plasmids matched best to the correct sequence (Supplementary Figure 1).

That the Ĉ-value for the reference sequence for pUUH239.2 is much lower than for R100 (Fig. 3b,c) was surprising since, theoretically, the Ĉ-value for the correct sequence should not decrease with increased plasmid size (Supplementary Methods 4). This unexpectedly low value suggested a possible discrepancy between the pUUH239.2 sample studied here and the theoretical pUUH239.2 reference sequence. A closer look at Fig. 2d reveals a striking difference between the experimental consensus barcode and the theoretical barcode in the region 180–220 kbp in pUUH239.2 (highlighted in Fig. 2d). This region contains the resistance cassette of the plasmid, composed of antibiotic resistance genes flanked by a number of transposable elements, most notably six identical copies of IS26-elements known to create a high frequency of rearrangements via homologous recombination^29^. Interestingly, the region of discrepancy between the theoretical and experimental barcodes in Fig. 2d is located in-between two inverted copies of IS26-elements, and a ~17 kb inversion was verified in the plasmid sample with directional PCR. Using a theoretical sequence of pUUH239.2 with an inversion of this region (Supplementary Figure 2), the Ĉ-value improved from 0.78 to 0.91 (Fig. 3c). Since structural variations are frequent in plasmids, we analyzed the minimum detectable size of structural variations by introducing random insertions, deletions or inversions into the theoretical sequence of R100 *in silico* and compared with the experimental consensus barcode. The results are shown in Supplementary Figure 3. How well a specific variation can be detected depends on the sequence information in that particular region and the plasmid size, but the main conclusion is that deletions and insertions with a size as small as 1 kbp lead to a significant decrease in Ĉ-value and are thus likely to be identifiable with our method.

### Evaluation of the assay *in-silico*

Since the barcode for a plasmid can be predicted from its theoretical sequence, we were able to reproduce the histograms in Fig. 3a–c for the corresponding theoretical barcodes. Comparing the Ĉ-value histograms for theory vs theory (Fig. 3d–f) to the histograms for experiment vs theory (Fig. 3a–c), we noted a strong resemblance. First, the Ĉ-values for the “non-match” barcodes decrease with increasing plasmid size, in agreement with theoretical predictions (Supplementary Methods 4). By “non-match” barcodes we refer to all theoretical barcodes except the correct sequence. It should however be noted that several of the “non-match” barcodes might contain *regions* with a high match to the barcode investigated. Furthermore, sequences that yield high Ĉ-values when compared with experimental consensus barcodes also yield high Ĉ-values when compared with the corresponding theoretical barcodes (Supplementary Figure 4). Our findings suggest that we can simulate the experimental Ĉ-value histogram for any sequenced plasmid. We therefore compared each theoretical barcode to all theoretical barcodes in the database to predict how well each plasmid can be separated from all other plasmids in the database. Fig. 4a shows the resulting Ĉ-values for the 3127 × 3127 pairs of sequenced plasmids; restricted to only include barcodes for plasmids that differ in size by less than 20%. The main observation is that the longer the plasmids are, the lower the Ĉ-values are for the non-match barcodes (brighter colors in the heat-map).

To transfer the information in Fig. 4a to experimentally realistic conditions we noted that the experimental Ĉ-value for the correct sequence is typically ~0.9 (see Fig. 3 above and Fig. 5 below). To be able to identify the correct sequence, a Ĉ-value of 0.9 thus has to be well separated from the Ĉ-values for the non-match barcodes. To quantify how this “separability” relates to plasmid size we fitted Gumbel distributions to all theoretical histograms and obtained scores of how well separated the correct sequence is from the non-match barcodes (see Fig. 3a–f, Methods and Supplementary Methods 6). We set two thresholds to estimate what plasmids would be possible to identify (Fig. 4b). The first threshold is based on Fig. 3a, where the correct sequence ends up on the very edge of the histogram (separability score of 0.01), but is not fully separated from the other sequences. The other threshold, one order of magnitude smaller (0.001), is for a separability score that defines a region where we are sure that the correct sequence yields a unique match (see also Supporting Figure S7 where we further elaborate on the uniqueness of a match). This analysis suggests that longer plasmids are easier to identify and that, using the less strict threshold, more than 90% of all plasmids between 70 and 300 kbp will be identifiable. The smallest plasmids that are possible to identify for a separability score of 0.01 are between 30 and 40 kbp. For plasmids larger than 300 kbp there are too few plasmids in the database to make the Gumbel fit statistically reliable but since the Ĉ-value for non-match barcodes decreases with plasmid size, such plasmids should also be possible to identify.

Figure 4b suggests that there is a significant dip in the capability of identifying plasmids around 180–190 kb. This dip is due to the fact that there are ~30 plasmids in this size regime with very high sequence similarity (theoretical Ĉ-values above 0.95) which leads to a lower separability score. However, the assay will readily detect this group of very similar plasmids, still yielding useful information regarding the plasmids studied.

### Characterization of plasmids in a clinical isolate

To show that the optical mapping assay also works on samples containing multiple different plasmids, and on clinical bacterial strains containing less characterized plasmids, we analyzed the plasmid content of an Extended Spectrum β-Lactamase (ESBL) producing *E. coli* strain, denoted isolate “ECO-005”, which was isolated from a patient with a urinary tract infection. Isolate ECO-005 was selected for this study since it has previously been investigated using traditional techniques and next generation sequencing, but the plasmid sequences were not completed and are therefore not yet included in the RefSeq database^30^. The earlier study concluded that isolate ECO-005 contains two plasmids, with sizes of 67 kbp and 139 kbp. The smaller plasmid, denoted plasmid pEC005A, contains the ESBL gene, while the larger plasmid, denoted plasmid pEC005B, contains resistance genes for aminoglycosides, trimethoprim, sulphonamides, tetracyclines, macrolides, and penicillins. The optical mapping assay confirmed that isolate ECO-005 contains two plasmids, with measured sizes in good agreement with the published sizes (Fig. 5a)^30^. When the experimental consensus barcode of pEC005A was matched to the database, a group of six plasmids (details in Supplementary Table 2) stood out with high Ĉ-values (Fig. 5b). The sequences of these six plasmids differ by less than 1% in their nucleotide composition and represent slightly divergent versions of a common ancestor. The previous study suggested that pEC005A is highly similar to plasmid pKF3-70 (NC_013542.1) from *Klebsiella pneumoniae*^30^, and this is also the plasmid that yields the highest Ĉ-value (~0.85) using our assay. The plasmid content of isolate ECO-005 was recently fully sequenced, yielding complete plasmid sequences (Sandegren, unpublished data). Compared to pKF3-70, the complete sequence of pEC005A differs by three insertions (1082, 828 and 72 bp) and two deletions (1639 and 244 bp). We created a theoretical barcode of the newly generated sequence for pEC005A, which yielded a significantly higher Ĉ-value (~0.93) than for any other plasmid in the database, again highlighting the resolution of the assay. For pEC005A, the assay has thus revealed a group of plasmids that are highly similar to the one studied as well as uniquely identified the specific plasmid.

When the experimental consensus barcode of pEC005B was compared to the database (Fig. 5d), the only well-separated Ĉ-value was for plasmid pAcX50e (NZ_CP010420.1, details in Supplementary Figure 6) with a relatively low Ĉ-value (0.70). A theoretical barcode was created from sequencing data on pEC005B and, again, the Ĉ-value was significantly higher than for any plasmid in the database (~0.87, Fig. 5e). The results for the two plasmids in ECO-005 show that we can predict best matches when a perfect match is not present in the database and also acquire an indication of the similarity based on the Ĉ-values.

## Discussion

The aim of this study was to demonstrate that optical mapping of single DNA molecules is a rapid and versatile tool for studying bacterial plasmids. By visualizing local variations in AT/GC content along stretched intact plasmids we are able to form *barcodes* with which plasmids can be identified using publically available sequence repositories. Furthermore, the very same experiments allow us to directly obtain the number and sizes of the plasmid types in the sample and thereby omit the need for time consuming traditional plasmid characterization methods, such as S1/PFGE analysis^25^. That the plasmids are inserted in their circular form and unfold to their linear form inside the channel guarantees that all plasmids analyzed are intact and that any other DNA is discarded.

The best fits of the experimental barcodes to the theoretical barcodes are obtained from consensus barcodes formed from several individual barcodes. This may be explained by two factors. Firstly, we minimize the inherent molecule-to-molecule variations in single-molecule experiments. Secondly, the ends of the individual barcodes are ill defined but since the plasmids were cut at random places along the contour, we eliminate this hurdle completely. The result is a circular barcode, with no ends, that covers the whole sequence of the plasmid. Our novel method for generating consensus barcodes can also be used to automatically separate plasmids of similar sizes into separate groups, as is discussed in detail in Supplementary Methods 5.

Our experimental results indicate that for plasmids ~70 kbp or larger, consensus barcodes can be used to statistically distinguish the correct plasmid sequence from a database of sequenced plasmids. Since this was based on a limited number of plasmids we confirmed our findings by comparison of all sequenced plasmids *in silico*. Smaller plasmids are less likely to be identified, since their barcodes contain less information, *i.e.* fewer peaks and valleys in fluorescence emission intensity, in agreement with our previous study^22^. It should be noted that our *in silico* study suggests that some plasmids as small as 30–40 kbp should be possible to identify. In this context it is important to remember that conjugative plasmids carrying resistance genes generally are larger than 50 kbp^10^.

Reference barcodes for plasmids not yet stored in the database can be generated both from optical mapping experiments and from calculating theoretical barcodes from sequencing data. As a result of the rapidly decreasing costs for DNA sequencing, the database is therefore continuously growing and will be significantly larger in the coming years, which will in turn improve the applicability and accuracy of our method.

Since the assay reveals holistic sequence information from intact plasmids, the assay is not sensitive to long repetitive sequences (e.g. transposons), which are difficult to resolve using existing DNA sequencing techniques due to limited read lengths (typically less than 1 kbp). Not only can this method replace DNA sequencing techniques in contexts where the barcodes provide sufficient data, but when detailed sequence information at a higher resolution is necessary, it can serve as the perfect complement to sequencing techniques by providing scaffolding via a “bird’s-eye” view. The combination of optical mapping and modern DNA sequencing has recently shown great promise in human genetics^19^,^31^; a similar potential for such a combination appears to exist for plasmid analysis.

An important aspect of the assay is its resolution, *i.e.* how large changes have to be in order to be detectable. We tackle this question by studying structural variations in plasmids R100 and pUUH239.2. Systematic *in silico* changes in the sequence of R100 revealed that changes down to 1 kbp should be possible to identify. For the ~220 kbp long plasmid pUUH239.2 we experimentally detected a ~17 kbp inversion compared to the publically available sequence that was not previously known. Although this identification was done “by eye”, we foresee the possibility of identifying such structural variations automatically, for example using tools similar to the ones developed by Marie *et al.*^32^.

Possible applications of our optical mapping assay for plasmid analysis range all the way from addressing fundamental questions in plasmid biology and dynamics to surveillance of antibiotic resistance plasmids in clinical settings. The assay is unique in the sense that it yields the size and the number of different plasmids in the isolate, along with sequence information that can identify previously sequenced plasmids. By inserting intact circular plasmids into nanofluidic channels, and visualize the transition from circular to linear configuration, we ensure that full plasmid sequences, and not plasmid fragments or remaining chromosomal DNA, are analyzed. This would not be possible in competing techniques, such as molecular combing, where the DNA is immobilized on a surface^13^. The barcode obtained will, for long enough plasmids, be a unique fingerprint of the plasmid of interest. With such a fingerprint it will be possible to identify plasmid transfer between bacterial strains and species as well as between patients even without any previous sequence information. This functionality is very important for surveillance of the spread of antibiotic resistance and should be of particular importance during nosocomial resistance outbreaks where it may shorten the time from sample collection to diagnosis substantially.

Importantly, our results imply that, for large enough plasmids, the barcode from a *single* plasmid molecule should be sufficient to identify it from the database. Hence, in terms of the amount of sample needed, we have reached the ultimate limit, where a single molecule is sufficient for analysis. This is in stark contrast to sequencing, where the average sequence of ~10^9^ bacteria is obtained. The minute amount of DNA needed suggests that it should be possible to use the assay directly on clinical samples, without culturing, which would reduce the time from sample collection to result significantly. All reagents used are commercially available and after extraction of plasmids, using standard kits, the remaining sample preparation is simple, requiring only standard pipetting. We have also developed software that automates all the steps involved in identifying a plasmid from recorded images. Nanofluidic channels can be made in plastic by injection molding, which could substantially reduce the cost of the nanofluidic devices used^33^. Furthermore, a recent paper from Wei, *et al.* demonstrated that single DNA molecules can be visualized using a compact and cost effective fluorescence microscope installed on a standard smartphone^34^, meaning that within the near future it will be possible to record optical DNA maps on smartphone cameras. This opens up the possibility to transfer the technology from the standard laboratory settings towards point of care analysis and diagnostics in, for example, developing countries. The optical mapping assay presented here is optimal for smartphone microscopy where there is a low photon budget since, in contrast to most other optical DNA mapping techniques, it is based on variations in a strong emission signal rather than emission from one, or very few, fluorophores.

To conclude, we demonstrate that optical DNA mapping is a versatile tool for characterizing and identifying bacterial plasmids. It complements existing techniques in many ways and is, to our knowledge, the first method for visualizing the intact sequence of plasmids. We foresee that this method has the potential to be a standard tool in microbiology labs around the world with applications ranging from standard microbiology to clinical diagnostics.

## Methods

### Plasmid preparation

Plasmid-containing strains were grown in Mueller Hinton broth at 37 °C overnight. Plasmid DNA was prepared from the over-night culture with the Qiagen Mini and Midi kits according to the manufacturer’s description for low-copy plasmids. Eluated DNA was precipitated with isopropanol, washed with 70% ethanol and resuspended in 10 mM Tris-HCl, 1 mM EDTA before analysis. The final concentration of eluated DNA was measured for each preparation using a NanoDrop.

### Sample preparation

For nanofluidics experiments, DNA was stained with YOYO-1 (YOYO, Invitrogen) in a molar ratio of 1:5 to the total number of basepairs in the sample, and with netropsin (Sigma-Aldrich) in a molar ratio of 150:1 with respect to YOYO. The samples were initially mixed in 5x TBE (Tris-Borate-EDTA, Medicago, diluted with ultrapure water from 10x tablets) and left to equilibrate at room temperature for about 20 minutes. As an example, 2 μL of plasmid DNA (100 μM, bp) was mixed with 2 μL λ-DNA (100 μM, bp), 2 μL of YOYO-1 (40 μM) and 3 μL of Netropsin (4000 μM). 5x TBE was then added in order to obtain a final volume of 10 μL. Subsequently, the samples were diluted to 0.05x TBE with ultrapure water to a final concentration of typically 0.4 μM of DNA (bp). The mixing in high ionic strength was performed to enable rapid equilibration of YOYO on DNA^35^. Beta-mercaptoethanol (BME, Sigma-Aldrich) was added in 2% (v/v) to suppress excessive photonicking of the plasmids. λ-DNA (48502 bp, New England Biolabs) was used as standard for measurements of the sizes of the plasmids and was measured in the same conditions as for the plasmids.

### Experimental procedure

**Figure 1:**
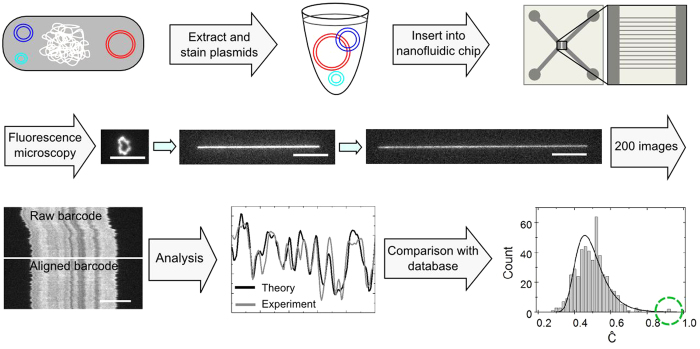
Workflow for the optical mapping assay for characterizing plasmids. Plasmids are extracted from bacteria, using standard commercial kits, and stained with YOYO-1 and netropsin in a single step to create a fluorescent barcode along the DNA. The sample solution is inserted into a nanofluidic chip and driven through the microchannels to the nanochannels with pressure (nitrogen gas). Intact circular plasmids are identified in the microchannel by eye and inserted into the nanochannels. The plasmid is irradiated with light until a double-strand break occurs, which causes the plasmid to unfold to its linear configuration and reveal the fluorescent barcode. Subsequently, ~200 images are taken of each single plasmid. Using automated software, the images are assembled into kymographs that are aligned to correct for fluctuations of the molecule inside the nanochannels. The aligned barcode is converted into an intensity trace along the plasmid. A database of theoretical barcodes based on known sequences is then constructed. Experimental barcodes are compared to this database and the sequence(s) with the best match to the barcode are identified (green circle). All scale bars correspond to 10 μm.

**Figure 2:**
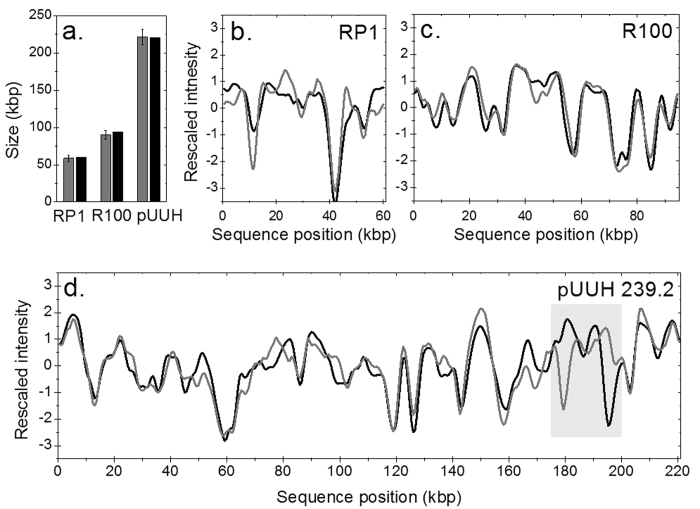
Optical mapping of bacterial plasmids. (**a**) Histogram of the average plasmid sizes of RP1, R100 and pUUH239.2, with nanofluidics data in gray and reference sizes from sequencing data in black. (**b-d**) Experimental consensus barcodes (gray) compared to the corresponding theoretical barcodes (black) for RP1 (**b**) R100 (**c**) and pUUH239.2 (**d**). The shaded area in (**d**) corresponds to the inverted region discussed in the text.

**Figure 3:**
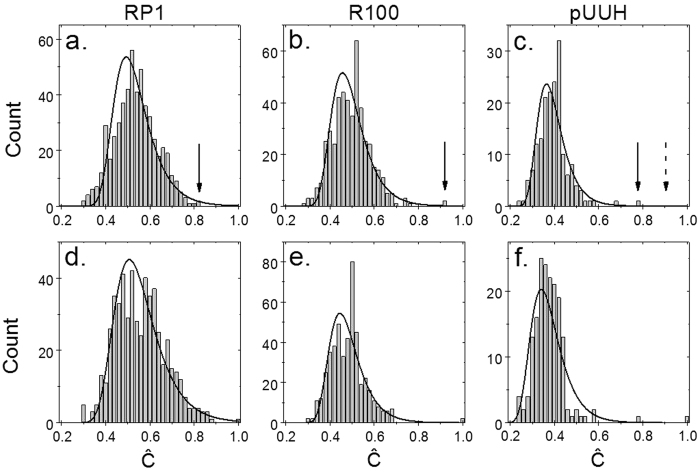
Comparing consensus barcodes with the database. (**a–f**) Histograms showing the best cross-correlation (Ĉ) when experimental consensus barcodes (RP1 (**a**), R100 (**b**) and pUUH239.2 (**c**)) and corresponding theoretical barcodes (RP1 (**d**), R100 (**e**) and pUUH239.2 (**f**)) are compared with theoretical barcodes for all sequenced plasmids in the public repositories within a size interval corresponding to ±3 standard deviations of the measured average sizes. Solid arrows mark the Ĉ-value of the correct sequence. The dashed arrow in (**c**) marks the Ĉ-value when the 17 kbp region has been inverted, see text. The solid lines are the Gumbel fits.

**Figure 4:**
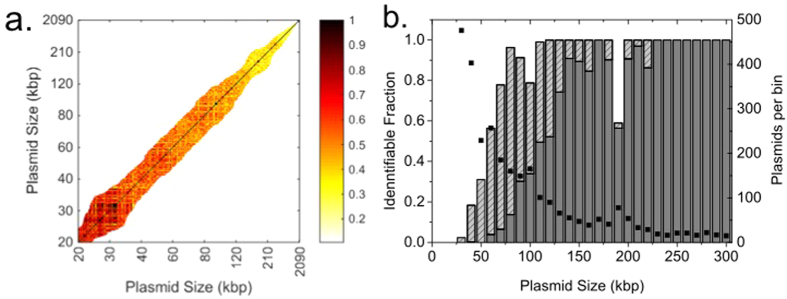
Comparing theoretical barcodes for all sequenced plasmids. (**a**) Ĉ-values for all pairs of plasmids within 20% in size. (**b**) Identifiable fraction of plasmids of a certain size. The dashed bars are based on a separability-score (0.01) that is on the limit of possible detection. The dark grey bars correspond to an order of magnitude lower separability-score (0.001). The bin size is 10 kbp and the number of plasmids per bin is shown on the right hand vertical axis (full squares).

**Figure 5:**
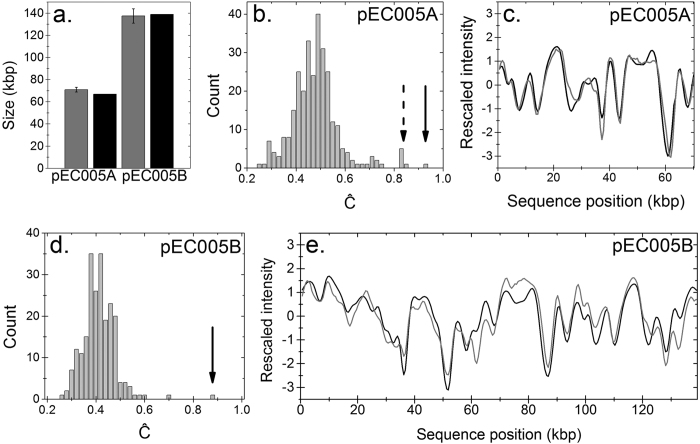
Characterization of plasmids from the clinical E. coli isolate ECO-005. (**a**) Size histogram with nanofluidics data in gray and S1/PFGE data in black. (**b**) Histogram of Ĉ-values for the experimental consensus barcode of pEC005A compared to all plasmids with a size corresponding to ±3 standard deviations of the measured average size of pEC005A. The black arrow marks the newly generated sequence, and the dashed arrow marks the six sequences that are highly similar to pEC005A, see text. (**c**) Experimental consensus barcode of pEC005A (gray) compared to the newly generated theoretical barcode (black). (**d**) Histogram of Ĉ-values for the experimental consensus barcode of pEC005B compared to all plasmids within a size interval corresponding to ±3 standard deviations of the measured average size of pEC005B. Black arrow marks the fit to the new sequence. (**e**) Experimental consensus barcode for pEC005B (gray) compared to the theoretical barcode of the new sequence (black).

Nanofluidic chips were fabricated in fused silica with standard methods as described elsewhere^15^. The nanofluidic chip consists of four loading wells connected two and two by microchannels, which in turn are spanned by nanochannels. The dimensions of the nanochannels were 100 × 100 nm^2^ or 100 × 150 nm^2^, both with a length of 500 μm. The channels were pre-wetted with a mixture of 0.05x TBE and 2% (v/v) BME. For each sample, a volume of 10 μL was loaded into the chip and the DNA was forced into the nanochannels by pressure-driven flow with nitrogen gas. All plasmids were inserted in their circular form, which was ensured by visual inspection of the plasmids in the microchannel, (see Fig. 1). The manual selection of intact circles ensures that all DNA molecules analyzed contain the entire sequence of the plasmid, and discards fragmented plasmids and remaining chromosomal DNA, which appear as linear pieces^24^. It should also be noted that nicking of the YOYO-labeled DNA will occur rapidly by irradiation with light and hence any supercoiled plasmids will be relaxed well before insertion into the nanochannels. Circular plasmids were unfolded to their linear form, while enclosed in the nanochannels, by spontaneous photonicking caused by irradiation with the excitation light of the microscope until a double-stranded break occurs^20^,^36^. The fact that all plasmids studied were linearized inside the nanochannel further guarantees that all plasmids analyzed are intact. After unfolding, the light was switched off and the molecules were allowed to relax into their equilibrium extension. Imaging was performed with an inverted fluorescence microscope (Zeiss AxioObserver.Z1) using a 100x oil immersion objective (Zeiss, NA = 1.46). Using an EMCCD camera (Photometrix Evolve) and an exposure time of 100 ms, a series of about 200 images was recorded for each molecule.

### Image analysis

Within our workflow, experimental barcodes are subject to three processing steps. First, raw kymographs (see Fig. 1) are aligned in order to reduce effects due to local conformational changes and center-of-mass diffusion of DNA during imaging, using the WPAlign method^37^. Once the kymograph has been aligned, we obtain the time-averaged barcode. Second, we remove the background from the signal region in the time-averaged barcode. To that end, we fit the sum of two sigmoidals to the data^25^. The estimated lengths, L, of the molecules are then obtained, and the barcode contains only the “signal region”. Third, we reduce end effects: in the end regions of experimental barcodes, the background and signal are intermixed due to the finite width of the microscope’s optical point spread function. To deal with this, and other end effects, we use a bit weighting scheme (Supplementary Methods 2) that effectively removes any effect due to the ends, yet retaining information about the true length, L, of the barcode.

### Generating consensus barcodes from experimental barcodes

As a result of stochasticity (for instance, due to the staining process) in individual experimental barcodes, time-averaged barcodes from DNA molecules with identical sequences differ slightly from one another. In order to reduce such molecule-to-molecule fluctuations, we present a new method for averaging *M* barcodes from multiple molecules with the same DNA sequence (but circularly shifted and possibly flipped) into “consensus” barcodes (Supplementary Methods 5). In brief, we first identify the two most similar barcodes out of all barcode pairs using a cross-correlation measure. These two barcodes are merged leaving us with *M-1* barcodes. The procedure is then repeated until the set of barcodes has been exhausted, resulting in a consensus barcode. In our procedure, a bit weighting method (see Supplementary Methods 2) reduces end effects and results in consensus barcodes which, in all cases considered, are truly circular (no edges).

### Theoretical barcodes

For comparisons with experimental data, we created a database of theoretical barcodes with sizes of 20 kbp and upwards. The sequences were retrieved from NCBI (http://www.ncbi.nlm.nih.gov/refseq/, June 2015) and filtered to remove all duplicates, and all sequences where more than 0.01% of the bases in the sequence are undefined, resulting in a database of 3127 sequences. The theoretical barcodes are created using the DNA sequence as input and are calculated using the known statistical physics framework for competitive binding of ligands^22^. The theoretical barcodes obtained from this framework have basepair resolution and are therefore convolved with the known point spread function of the microscope in order to mimic experimental conditions.

### Identifiability of plasmids

To address the question whether a particular plasmid can be experimentally identified in the database, we apply a two-step strategy: (I) First, if the correct plasmid’s length differs more than 20 percent in size from another plasmid, these two plasmids are deemed different. (II) For barcodes in the database of similar length to the correct plasmid, see criteria (I), we introduce a separability score (details below). If this score is smaller than a predetermined threshold then the correct plasmid is said to be identifiable. To calculate the separability score we first quantify the similarity of the barcode of the correct plasmid (experimental or theoretical) with all K theoretical barcodes of similar lengths, using best Pearson correlation coefficient, *Ĉ*_*k*_, k = 1,….K. The term “best” here refers to the fact that, due to the circular nature of plasmids, it is necessary to slide one barcode across the other, and possibly to flip it. The sliding generates a set of cross-correlation values out of which only the best one is stored. Note that in general cross-correlation values are in the range of -1 to 1. However, the “best” cross correlation is generally in the range of 0 to 1. In all our studied cases all Ĉ-values are indeed positive. For large enough K, these best cross-correlation values are expected to follow the Gumbel probability density function (PDF), *Φ(Ĉ)* (probability density for the best, with two parameters)^38^ and we therefore fit the histogram of *Ĉ*_*k*_ to the Gumbel PDF. In the Results section we find that a match between an experimental plasmid barcode and its “correct” theoretical barcode has *Ĉ* ≈ *Ĉ*_*match*_ = 0.9. Therefore, once the two Gumbel parameters have been estimated we define a separability score, which is similar to the usual p-value definition, *i.e.*, the separability score equals 
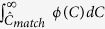
. By construction, the separability score is in the range 0 to 1 and has an average score of 0.5. We deem a plasmid identifiable if the separability score is smaller than a set threshold. In the Results section we use a conservative threshold of 0.001 and a less restrictive choice, with a threshold of 0.01, and demonstrate that the plasmid identifiability is insensitive to this choice for long enough plasmids (>70 kbp). Details are found in Supplementary Methods 6.

## Additional Information

**How to cite this article**: Nyberg, L. K. *et al.* Rapid identification of intact bacterial resistance plasmids via optical mapping of single DNA molecules. *Sci. Rep.* **6**, 30410; doi: 10.1038/srep30410 (2016).

## Supplementary Material

Supplementary Information
