# Corrigendum: Rapid identification of intact bacterial resistance plasmids via optical mapping of single DNA molecules

**DOI:** 10.1038/srep46911

**Published:** 2017-12-22

**Authors:** Lena K. Nyberg, Saair Quaderi, Gustav Emilsson, Nahid Karami, Erik Lagerstedt, Vilhelm Müller, Charleston Noble, Susanna Hammarberg, Adam N. Nilsson, Fei Sjöberg, Joachim Fritzsche, Erik Kristiansson, Linus Sandegren, Tobias Ambjörnsson, Fredrik Westerlund

Scientific Reports 6: Article number: 30410; 10.1038/srep30410 published online: 07 26 2016; updated: 12 22 2014.

In the Supplementary Information file originally published with this Article, Figures S8 and S12 were omitted.

These errors have been corrected in the Supplementary Information file that now accompanies the Article.

