# Supplementary material for: Rapid identification of intact bacterial resistance plasmids via optical mapping of single DNA molecules
Source: Sci Rep. 2016 Jul 27;6:30410. doi: 10.1038/srep30410 (PMC4961956; doi:10.1038/srep30410)
Supplement: Supplementary Information [file srep30410-s1.pdf]

Supplementary Information: Rapid identification of intact bacterial resistance plasmids via optical mapping of single DNA molecules

Lena K. Nyberg,[1] Saair Quaderi,[1,2] Gustav Emilsson,[1,3] Nahid Karami,[4] Erik Lagerstedt,[2] Vilhelm Müller,[1] Charleston Noble,[2] Susanna Hammarberg,[2] Adam N. Nilsson,[2] Fei Sjöberg,[4] Joachim Fritzsche,[3] Erik Kristiansson,[5] Linus Sandegren,[6] Tobias Ambjörnsson,[2] Fredrik Westerlund[*1]

[1]Department of Biology and Biological Engineering, Chalmers University of Technology, Gothenburg, Sweden
[2]Department of Astronomy and Theoretical Physics, Lund University, Lund, Sweden
[3]Department of Applied Physics, Chalmers University of Technology, Gothenburg, Sweden
[4]Department of Infectious Diseases, Sahlgrenska Academy, University of Gothenburg, Gothenburg, Sweden
[5]Department of Mathematical Sciences, Chalmers University of Technology/University of Gothenburg, Gothenburg, Sweden
[6]Department of Medical Biochemistry and Microbiology, Uppsala University, Uppsala, Sweden

S.T Supplementary Tables

Supplementary Table 1: Reference data and experimental data on the plasmids investigated

Reference data		Experimental data			
Plasmid	Size (kbp)	Measured size (kbp)[b]	Size interval used[c]	Number of experiments[d]	Number of theoretical sequences[e]
RP1	60.1	58.5 (4.0)	46.5-70.5	18	539
R100	94.3	90.0 (5.6)	73.2-106.8	25	471
pUUH239.2	220.8	221.3 (10.3)	190.4-252.2	14	174
pEC005A	67[a]	71.0 (2.1)	65-77	13	295
pEC005B	139[a]	137.6 (6.4)	118-157	14	222

[a]From PFGE data.
[b]Standard deviation in parenthesis
[c]The size interval used when the experimental consensus barcodes are compared with the sequences in the database.
[d]The number of individual barcodes used to form the consensus barcode.
[e]The number of theoretical sequences in the database in the specified size interval.

Supplementary Table 2: Names and sizes of the six highly similar plasmids that gave high $\hat{C}$-values for the comparison with the consensus barcode of plasmid pEC005A

Plasmid	Reference Sequence	Size (kbp)
pHK08	NC_019072.1	69.8
pKF3-70	NC_013542.1	70.1
pHK17a	NC_016039.1	70.1
pHK01	NC_019057.1	70.3
pEG356	NC_013727.1	70.3
pHK09	NC_019071.1	70.4

S.F Supplementary Figures

S.F.1 Individual barcodes

Figure S1a shows that for the larger plasmids R100 and pUUH239.2, a majority of the individual barcodes will allow identification of the correct plasmid from the database. Figure S1b-d show individual experimental barcodes of RP1, R100 and pUUH239.2 compared to the theoretical sequence, showing excellent overlap.

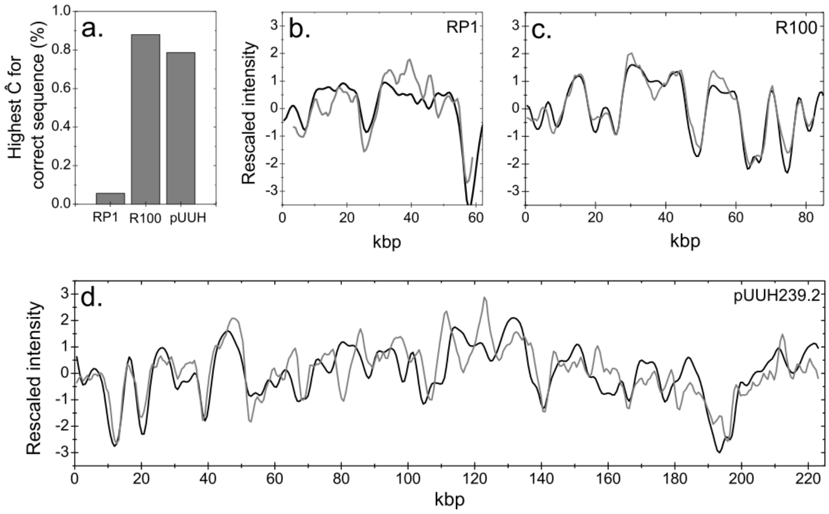

Figure S1: Individual barcodes. (a) Percentage of the individual barcodes that have the highest $\hat{C}$-value for the correct theoretical sequence, when compared to all sequences within 3 STD of the measured mean size. (b-d) Experimental individual barcodes (gray) compared to the corresponding theoretical barcodes (black) for RP1 (b), R100 (c) and pUUH239.2 (d).

S.F.2 pUUH239.2 with inversion

Figure S2 shows the experimental consensus barcode of pUUH239.2 compared to the theoretical barcode with the inversion discussed in the main text.

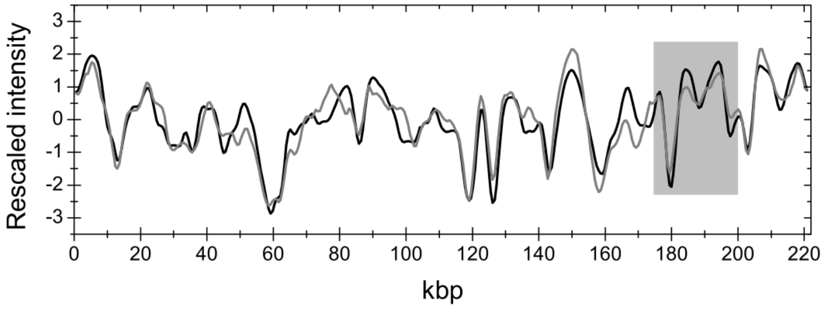

Figure S2: pUUH239.2 with inversion. Consensus barcode (gray) compared to the theoretical barcode with the inversion (black) for pUUH239.2.

S.F.3 Testing assay resolution

To test the resolution of the assay, we decided to modify the theoretical sequence of R100 and compare it to the corresponding experimental consensus barcode. We choose four different modifications to the sequence:

1. Deletion: where a subsequence is removed
2. Duplication: where a subsequence is repeated as an insertion
3. Inversion: where a subsequence is inverted
4. Transposition: where a subsequence is moved to another location

For each type of modification of a given length, we applied the modification in 10 separate instances at randomly chosen locations in the sequences. We increased the size of the modification from 250 bp to 17 kbp. For deletions, duplications, and transpositions we observed significant decreases in $\hat{C}$ when the modifications were larger than 1 kbp. Inversions had less impact on the maximum correlation coefficients making them more difficult to detect.

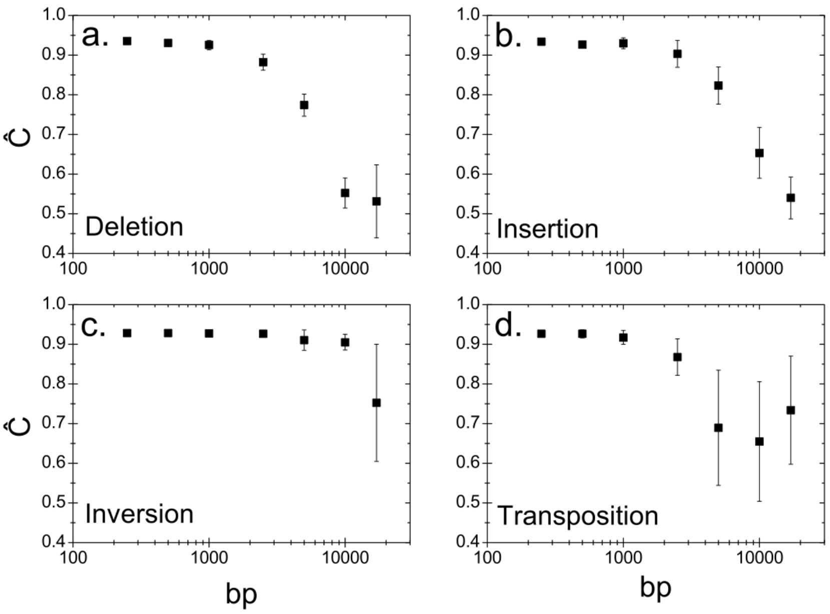

Figure S3: Structural variations. Maximum correlation coefficient of the experimental consensus barcode of plasmid R100 when compared to modified theoretical barcodes of R100 with deletions (a), insertions (b), inversions (c), and transpositions (d) of increasing sizes.

S.F.4 Comparing theory vs theory and theory vs consensus

In the main text are histograms of theory versus theory (TvT) and theory vs consensus (TvC), see Figure 3 in the main text. We noticed the great similarity for the TvT and TvC results. To further elaborate on this similarity, Figure S4 shows the "raw" data underlying the histograms in the main text, namely the maximum correlation coefficients for R100, RP1 and pUUH239.2 as a function of length of the plasmid to which these three plasmids are compared (a-c) and as scatter plots (d-f) where, even though the correct sequence has a lower value than 1 (due to experimental reasons, as discussed in the main text), the comparison shows a narrow distribution of the majority of the $\hat{C}$-values around y = x.

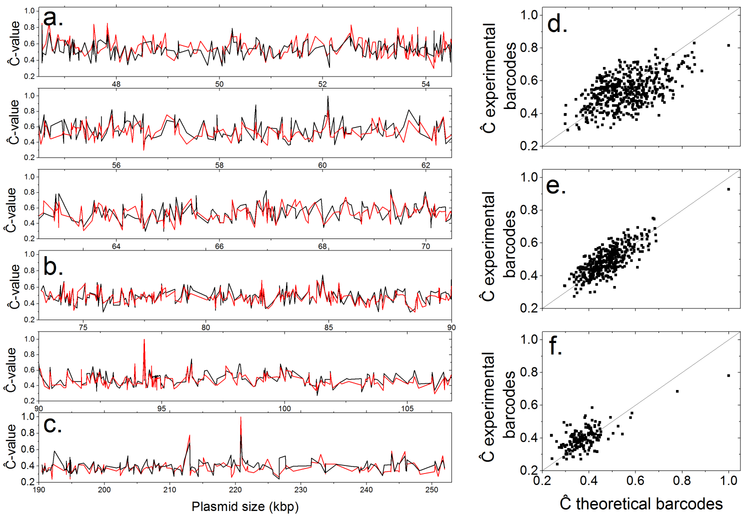

Figure S4: **Comparison of plasmid pairs in theory database and theory vs three experimental consensus barcodes.** Theory vs theory and theory vs consensus plots for the RP1 (a, d), R100 (b, e) and pUUH239.2 (c, f) plasmid as a function of plasmid length (a-c, red = theory, black = consensus) and as a scatter plot where black line corresponds to y=x.

S.F.5 The variation of $\hat{C}$ for theory vs theory with plasmid length

In order to evaluate the general applicability of DNA optical maps for differentiating between plasmids, in the main text we calculated theoretical barcodes for all the 3127 sequenced plasmids. The similarity of all TvT pairs were compared using a maximum correlation coefficient matrix, $\hat{C}_{ij}$ (see Figure 4a in the main text). We now provide some supplmentary post-processing results of $\hat{C}_{ij}$. Figure S5 displays the mean cross correlation and associated standard deviation by averaging over each row of the matrix as a function of plasmid size. We see that the mean maximum correlation coefficient decays with plasmid size. This results is in qualitative agreement with the main finding in equation S5 (below) which thus has importance also for "real" barcodes. In contrast to the mean, the standard deviation around the mean does not have a clear monotonic dependence on the plasmid size.

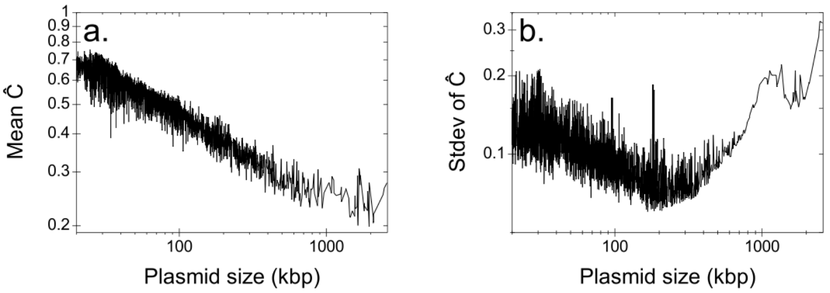

Figure S5: **The mean and the standard deviation of the maximum correlation coefficient depend on plasmid length.** Mean and variance of each row in the theory vs theory maximum cross correlation matrix displayed in Figure 4a in the main text. The “whitened-out” regions are not included. Notice that the mean maximum correlation coefficient systematically decrease with length of the barcodes.

S.F.6 Comparison of pEC005B and pAcX50e

Figure S6 shows a comparison of the theoretical barcode of pEC005B and the sequence with the second best fit to pEC005B, plasmid pAcX50e. Note that the theoretical barcodes are similar in their overall shape but on the local level they differ significantly in agreement with that they have no sequence similarity.

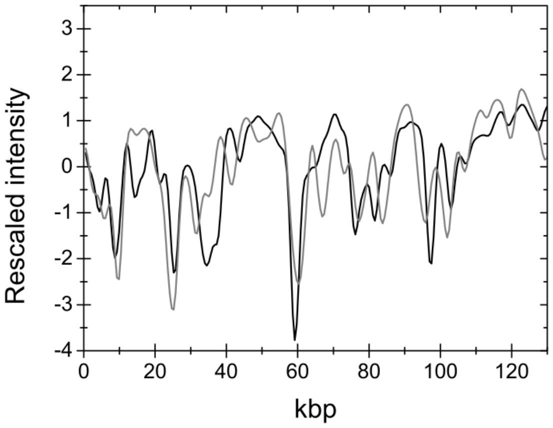

Figure S6: Comparison of the theoretical barcode of plasmid pAcX50e (black) and the theoretical barcode of 005B (gray).

S.F.7 Uniqueness of match

In the main text we found that, generally, plasmid barcodes > 70 kbp can be identified from the theory barcode database (using the experimental "match"-condition, $\hat{C} > 0.9$). In Figure S7 we show *how many* plasmid barcodes > 70 kbp which end up in the tail of the fitted Gumbel PDF (at significance levels $\alpha = 0.01$ (top) and 0.001 (bottom), non-recursive fitting). We find that in a significant fraction of the cases, the correct plasmid is the only plasmid in the tail of the Gumbel PDF (unique "match").

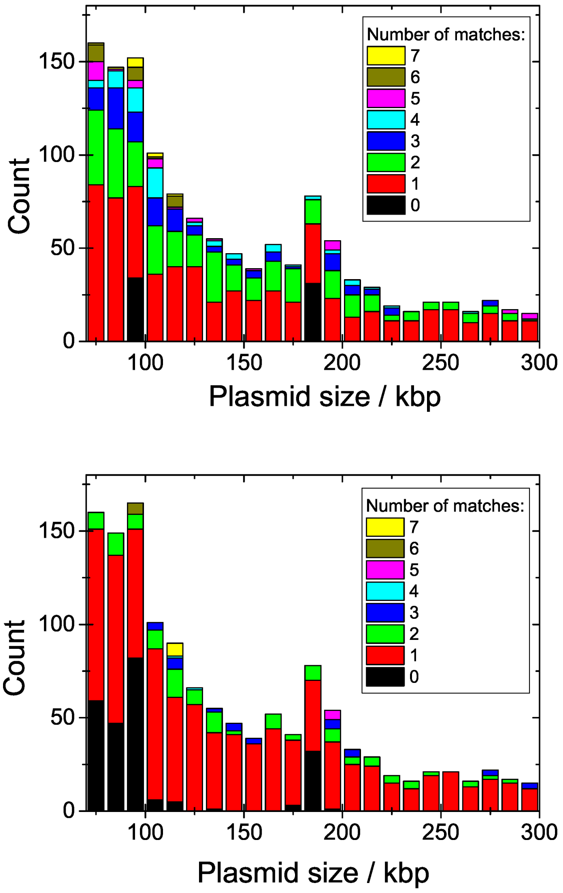

Figure S7: **Uniqueness of "match".** Histograms of the number of plasmid barcodes in the tail of fitted Gumbel PDF, at significance levels $\alpha = 0.01$ (top) and $\alpha = 0.001$ (bottom), respectively.

S.M Supplementary Methods

S.M.1 Generating theory barcodes from sequences

Theoretical plasmid barcodes from a database are used extensively throughout the main text. The database contains theoretical barcodes for all of the 3127 unique plasmid DNA sequences in the database, providing data in a format against which experimental data can be compared. Theory barcodes are calculated using the known statistical physics framework for competitive binding of ligands; see [2, 3, 4]. In brief, the current setup uses two ligands, netropsin and YOYO-1. Both of these molecules occupy four basepairs when bound to DNA. Netropsin binding constants are sequence-specific whereas YOYO-1 binds non-specifically. By formulating the statistical physics problem of where these ligands are likely to bind in terms of transfer matrices, and obtaining values for relevant binding constants from the literature, we were able to establish

an avenue to numerically calculate the probability, $p(i)$, that a base-pair i, is occupied by (one of the monomers of) a YOYO-1 ligand.[4] Note that the probability, $p(i)$, has basepair resolution. In contrast, experimental data comes in the form of a significantly lower resolution barcode (due to the limiting optical microscope resolution) obtained at pixel level. Furthermore, in experiments, DNA molecules are not fully extended in the nanochannels. To account for these differences when comparing theory and experiment, experimental conditions are simulated by first translating the quantity i into length (μm) and then convolving a point spread function in the shape of a Gaussian with $p(i)$. Finally, the theoretical barcode may be sampled at points separated by a fixed interval length to produce barcodes that are of a given length in "pixel resolution" such that it can be directly compared to an experimental barcode. Details are found in [4].

S.M.2 Handling of individual experimental barcodes

Experimental data must undergo a few preprocessing steps before it can be compared to theoretical barcodes. These steps are described in the three subsections below.

S.M.2.1 Kymograph alignment

DNA molecules are subject to local conformational changes and center-of-mass diffusion as they are imaged using the barcoding method as described in the main text. Therefore, before time-averaged individual barcodes, which we use throughout our study, can be extracted from experimental data (kymographs), the effect of noise is reduced by aligning the features in the kymograph. This is achieved using a computationally fast algorithm called WPAlign[1]. Once the kymograph has been aligned we calculate an average over the "rows" in the kymograph in order to obtain an individual time-averaged experimental barcode.

S.M.2.2 Identifying the edges of DNA barcodes

Time-averaged barcodes contain some background with lower intensity in addition to the fluorescence signal from the DNA molecule (high intensity region); see example in Figure S8. In order to identify a "start" and "end" point of the molecular data in a barcode, we fit it to a function $g(x)$

$$g(x) = a + (\tanh((x-b)\,d) - \tanh((x-c)\,e))\,f. \tag{S.1}$$

using a least squares approach, where the fitting parameters a-f are adjusted to minimize the sum of squared residuals. The parameters b and c specify the start and end points of the barcode. The initial guess values for b and c are set to the first and last zero-point crossings obtained by subtracting the mean of the signal from the data signal. The function $g(x)$ fitted to an R100 plasmid barcode is shown in Figure S8. The estimated length of the "signal region" representing the molecule is $L \approx c - b$. The barcode $B(i)$ is defined over indices from $0 \leq i \leq L-1$ with the values of the pixels from b through c (inclusive after rounding b and c to the nearest integer pixel indices).

Figure S8: **Method for edge detection in experimental barcodes.** A time-averaged experimental barcode consists of a signal region in the middle of two background regions. The function $g(x)$, equation (S.1), is fitted to the time-averaged experimental barcode data. The fitting parameters for the start and end points, b and c, which represent the detected edges of the molecule, are indicated as vertical lines.

S.M.2.3 Bit-weighting edge regions

Experimental DNA barcodes, such as the barcode shown in Figure S8, have particular distortions and fluctuations at their edges, which could introduce misleading data that adversely affects downstream analysis if they are not accounted for. There are three main reasons for these distortions:

1. the experimental signal is "filtered" by the optical point spread function PSF (with a width, w, on the order of 300 nm); this filtering makes it likely for barcode end regions to contain a mixture of background and actual signal over distances of w
2. DNA molecules have more pronounced local conformational fluctuations at their ends compared to interior parts
3. the kymograph alignment algorithm, WPAlign [1], used herein, does not explicitly align the starts and ends together, so greater mixtures of background with actual signal are likely to be present at the end regions

Here we introduce a simple "bit-weighting" scheme which allows us to avoid many of the undesirable effects that can arise from distortions at edges that have not been taken into account. Similarly to $B(i)$, we define bit weights $W(i)$ for all pixels in the region $0 \leq i \leq L-1$. In our implementation, the weight for a pixel i, $W(i)$, which is associated with a barcode value $B(i)$ are simply defined: a pixel is given a weight of 0 if it is in an "end region" and a weight of 1 otherwise. End regions are defined as the regions where the distance from the index 0 and $L-1$ is less than or equal to a predefined length of an end region, Δ.

$$W(i) = \begin{cases} 0 & \text{if } (0 \leq i \leq \Delta - 1) \text{ or } (L - \Delta \leq i \leq L-1) \\ & \text{(i.e pixel is in an "end region")} \\ \\ 1 & \text{if } (\Delta \leq i \leq L - \Delta - 1) \end{cases} \tag{S.2}$$

In this work, we have defined $\Delta = rw$ with $r = 3$. The bit weights produced are utilized in Secs. S.M.4 and S.M.5 in the processes of comparing and merging experimental barcodes in order to suppress the negative influence of the problematically uncharacteristic data that tends to be present at the end regions.

S.M.3 Length rescaling of barcodes

In our study, we handle barcodes under the premises that they are circular with a period of their detected length. In many cases, we wish to compare

the similarity of two barcodes by appealing to pairwise comparisons of their values. If we have two circular barcodes of differing lengths, these comparisons are complicated by the need to rescale them to a common length. The bit-weights associated with the barcodes must also be scaled to, or produced, at that common length. To scale the barcodes we first determine a target length for the barcodes and then sample or interpolate the barcodes as necessary to produce versions of the barcodes which have a common length. For our study, there were three scenarios under which a design choice was made regarding how to rescale the barcodes. We are able to produce bit-weights after the rescaling, so bit-weights did not have to undergo any sampling or interpolation. Here we describe the scenarios and our approaches to handling them:

1. When generating a consensus barcode, the target length chosen for the rescaled barcodes was the mean of the lengths for the individual barcodes being rescaled, rounded to the nearest integer. For the length rescaling we use sampling and linear interpolation. The bit-weights that specified end regions on the rescaled barcodes were simply computed in accordance with the resized length. The bit-weights for the rescaled barcodes were generated based on the same approach of setting values of 0 for a fixed number of pixels in the end regions as is described in Sec. S.M.2.3. The algorithm for calculating the values for a consensus barcode and its corresponding bit-weights are detailed in Sec. S.M.5.

2. When comparing a theoretical barcode to an experimental barcode, the target length chosen was that of the experimental barcode so that the experimental data did not have to be sampled/interpolated and that its bit-weights could be left alone. The bit-weights for the theory are all 1 since they have no distorted end regions so the change in size also requires no extra work in terms of adjusting bit-weights.

3. When comparing a theoretical barcode to another theoretical barcode, the first theoretical barcode was adjusted to the size of the second barcode. All bit-weights were set to 1. This approach was chosen so that results for each pair of two theoretical barcodes would be more comparable to results for each pairing of a theoretical barcode with an experimental consensus barcode.

S.M.4 Quantifying barcode similarity

In this section, we define and analyze our similarity score, $\hat{C}$.

S.M.4.1 Parameterizing barcode alignment

Consider a pair of barcodes, B_1 and B_2, where B_1 is a circular barcode with a length N_1 which is no shorter than the length of B_2, N_2. Our similarity score, $\hat{C}$, is semantically defined to represent the maximum Pearson correlation coefficient that can be produced by comparing B_1 and B_2 across an exhaustive set of valid "alignment possibilities" for the sequential values in B_1 and B_2.

The possible reorientation configurations can be understood through and expressed by two parameters:

1. a relative "flip" parameter, f: The flip parameter is used to account for the fact that the directions of the DNA molecules associated with the two barcodes may be either the same as one another ($f = 1$) or the opposite of one another ($f = -1$).

2. a relative circular shift (i.e. periodic delay) parameter, d, defined as an integer in *modulo* N_1. The parameter d is used to explore each of the N_1 different indices along B_1 which the first index of the sequence of values in barcode B_2 can be shifted to in order to produce a new pairing (i.e. alignment) for their (ordered) values.

There are thus a total of $2N_1$ valid alignments that can be parameterized by pairs of f and d.

S.M.4.2 Pearson correlations of barcodes with weights

Using the pairing of barcode values at a combination of f and d, we compute a Pearson correlation coefficient, $C(f, d)$. Pearson correlation measures linear correlation for two variables, where the coefficient values produced are between -1 and 1, the expected value for uncorrelated data is 0, and a value of 1 denotes a total perfect correlation (e.g. a perfect match).

We now define

$$\hat{C} = \max\{C(f, d)\} \tag{S.3}$$

as our similarity score between two circular barcodes, and note that this maximum generally has an expected value > 0 even for uncorrelated data. The manner in which this expected value for $\hat{C}$ is expected to scale with a sample size of N is elaborated in subsections S.M.4.5 and S.M.4.6.

Herein, the values in our two barcodes, B_1 and B_2 are also associated with "bit-weights" provided in W_1 and W_2. The rationale for these bit-weights is discussed in Sec. S.M.2.3. The bit-weight associated with a value in a barcode represents whether the value may be included when computing the Pearson correlation coefficient or not. In the calculations of Pearson correlation coefficients, at a given alignment produced by a pair of f and d, any pairs of values in B_1 and B_2 which are associated with a weight of 0 in either W_1 or W_2 would be excluded. Any values in B_1 which are unpaired with any value due to some difference in length where $N_2 < N_1$ are excluded.

In Sec. S.M.4.3, we explore the mathematics behind the efficient computation of $C(f, d)$ for circular barcodes B_1 and B_2 with bit-weights provided in W_1 and W_2, assuming that their lengths are equal ($N = N_1 = N_2$). Our barcode preprocessing step described in Sec. S.M.3 explains how we arrive at barcodes of equal length for this study.

Although we do not deal with fragments in our study of circular barcodes, it may be interesting to note that if $N_2 < N_1$, and B_2 represents a potential fragment of a circular barcode of length N_1, one can simply "zero-pad" the barcode B_2 and its bit-weights W_2 with extra zeros at their end until they also have a length of N_1 without affecting the values of $C(f, d)$. This can be understood by considering the fact that all values for B_2 with weights of 0 will always be excluded from the samples and that padding the shorter barcode at the end would not distort the alignment of values for the barcodes in any undesirable way. Thus, the requirement for equal length does not result in a

loss of generality for the methodology described in Sec. S.M.3 if it is to be used on a circular barcode of length N_1 and a smaller fragment of a circular barcode with length $N_2 < N_1$, since B_2 and W_2 can trivially be extended to meet the requirement.

S.M.4.3 FFT friendly representation of $C(f, d)$

Here, we present a mathematical representation of the Pearson correlation coefficient parameterized by f and d [equation (S.4)] for a pair of barcodes (B_1 and B_2) with bit-weights (W_1 and W_2), all of a common length, N. This formulation allows us to demonstrate how we may efficiently compute the coefficient for all pairs of f and d, allowing us to find the maximum Pearson correlation coefficient, [equation (S.3)]. The bit-weighted cross correlation used throughout this study is defined:

$$C(f,d) = \frac{p_{12}(f,d) - n(f,d)\,\mu_1(f,d)\,\mu_2(f,d)}{\sqrt{\left(p_{11}(f,d) - n(f,d)\,(\mu_1(f,d))^2\right)\left(p_{22}(f,d) - n(f,d)\,(\mu_2(f,d))^2\right)}}. \tag{S.4}$$

This definition is a composite of a set of functions [see equations (S.8)-(S.13)] parameterized by f and d, which will be addressed shortly, but first, let us define weighted barcodes $B_j^\star$ for $j = 1, 2$ as follows:

$$B_j^\star(i) = W_j(i)\,B_j(i) \tag{S.5}$$

Let us also denote the squares of the weighted barcodes as $B_j^{2\star}$ noting also that $W_j(i) = (W_j(i))^2$ since bit-weights are 0 or 1.

$$B_j^{2\star}(i) = (B_j^\star(i))^2 = W_j(i)\,(B_j(i))^2 \tag{S.6}$$

The sample means for weighted barcodes $j = 1, 2$ with a given shift d and flip f after excluding values that correspond to bit-weights of 0 are given by $\mu_j(f, d)$ [equation (S.7)].

$$\mu_j(f,d) = \frac{s_j(f,d)}{n(f,d)} \tag{S.7}$$

where the effective sample size, $n(f, d)$, is defined in equation (S.8) and $s_j(f, d)$ is defined in equations (S.9) and (S.10). If half or more of the bit-weights in W_1 or W_2 are 0, the equations (S.4) and (S.7) could contain a division by 0. This situation never occurred in any of our calculations, but if the denominators were to equal 0, the values for $C(f, d)$ and $\mu_j(f, d)$ could be considered undefined and represented as NaN.

The values for all the other new functions represent simple sliding dot-products (i.e. unnormalized cross-correlations) that may be computed at all the circular shifts and flips in linearithmic time $\mathcal{O}(N \log N)$ using the fast Fourier transform (FFT) algorithm. To do so, the task can be converted into a circular convolution problem by flipping either barcode. The circular convolution can be performed linearithmically by converting the barcodes to the frequency domain with FFTs, performing element-wise multiplication and converting the results back to the time domain with an inverse fast Fourier transform (IFFT).

With a given flip (f) and shift (d), the sample size is provided by $n(f, d)$ [equation (S.8)]. For weighted barcode $j = 1, 2$ the sums of the samples are given

by $s_j(f,d)$ [equations (S.9) & (S.10)], the sums of the squares of the samples are given by $p_{jj}(f,d)$ [equations (S.11) & (S.12)], and finally, the sums of the products of the paired samples are given by $p_{12}(f,d)$ [equation (S.13)]. For simplicity, we denote the flipped versions of W_2, $B_2^\star$, and $B_2^{\star 2}$ with a subscript index of -2 (instead of 2) such that W_{2f}, $B_{2f}^\star$, and $B_{2f}^{\star 2}$ refer to the original versions when $f=1$ and to the flipped versions when $f=-1$. To be precise, the "flipping" of a barcode simply means that for an index i, the new value associated with it should be the old value for the barcode at index $(N-i) \pmod N$.

$$n(f,d) = \sum_{i=0}^{N-1} W_{2f}(i)\, W_1((i-d) \pmod N) \tag{S.8}$$

$$s_1(f,d) = \sum_{i=0}^{N-1} W_{2f}(i)\, B_1^\star((i-d) \pmod N) \tag{S.9}$$

$$s_2(f,d) = \sum_{i=0}^{N-1} B_{2f}^\star(i)\, W_1((i-d) \pmod N) \tag{S.10}$$

$$p_{11}(f,d) = \sum_{i=0}^{N-1} W_{2f}(i)\, B_1^{2\star}((i-d) \pmod N) \tag{S.11}$$

$$p_{22}(f,d) = \sum_{i=0}^{N-1} B_{2f}^{2\star}(i)\, W_1((i-d) \pmod N) \tag{S.12}$$

$$p_{12}(f,d) = \sum_{i=0}^{N-1} B_{2f}^\star(i)\, B_1^\star((i-d) \pmod N) \tag{S.13}$$

S.M.4.4 Summation representation of $C(f,d)$

While the formulation for $C(f,d)$ presented in Sec. S.M.4.3 is relatively straightforward and especially efficient for computations, for the purposes of normalizing $\hat{C}$ with respect to its expected value for a given N as will be discussed in Secs. S.M.4.5 & S.M.4.6, it is useful to present $C(f,d)$ in the form of the summation in equation S.14.

$$C(f,d) = \frac{1}{n(f,d)-1} \sum_{i=0}^{N-1} \tilde{B}_1(f,d,i)\, \tilde{B}_2(f,d,i) \tag{S.14}$$

For barcodes $j=1,2$, the quantities $\sigma_j(f,d)$ (equations (S.15) & (S.16)] provide the (unbiased) standard deviations for the samples (with a given flip f and shift d):

$$\sigma_1^2(f,d) = \frac{1}{n(f,d)-1} \sum_{i=0}^{N-1} \left(B_{2f}^\star(i)\, W_1((i-d) \pmod N) - \mu_1(f,d)\right)^2 \tag{S.15}$$

$$\sigma_2^2(f,d) = \frac{1}{n(f,d)-1} \sum_{i=0}^{N-1} \left(W_{2f}(i)\, B_1^\star((i-d) \pmod N) - \mu_2(f,d)\right)^2 \tag{S.16}$$

Now, for barcodes $j = 1, 2$, $\tilde{B}_j(f, d, i)$ [equations (S.17) & (S.18)] represent the values of the barcodes at index i after all the samples in the barcode have been linearly rescaled to have a new sample mean of 0 and a sample standard deviation of 1 (with a given flip f and shift d):

$$\tilde{B}_1(f, d, i) = \frac{(W_{2f}(i) B_1^{\star}((i - d) \pmod N)) - \mu_1(f, dF))}{\sigma_1(f, d)} \tag{S.17}$$

$$\tilde{B}_2(f, d, i) = \frac{(B_{2f}^{\star}(i) W_1((i - d) \pmod N)) - \mu_2(f, d))}{\sigma_2(f, d)} \tag{S.18}$$

With these definitions, we are able to form a representation of $C(f, d)$ in the summation representation provided in equation (S.14).

S.M.4.5 Expected scaling of $\hat{C}$ for non-matching barcodes with respect to N

In the main text (see also Figure S5) we find that when comparing a plasmid barcode of length N to a non-matching (i.e. unrelated) plasmid barcode of similar length, the corresponding maximum correlation coefficient value decreases with N. In this subsection we provide an explanation for this finding based on a derivation of the expected N-dependence of the maximum correlation coefficient when matching two random barcodes to each other.

Consider again our choice of similarity score, the correlation coefficient, as expressed in equation (S.14), and assume that the barcodes $B_1(i)$ and $B_2(i)$ are independent random numbers picked from the same probability density function (PDF). Denote the sample size by N. If $N \gg 1$, then equation (S.4) is a sum of many independent, identically distributed, random numbers with mean zero, allowing us to apply the central limit theorem to find that the PDF for the correlation coefficients becomes a Gaussian [equation (S.19)] with mean zero and variance S [equation (S.20)] where γ is the variance for the random numbers expressed by $\tilde{B}_1(f, d, i) \tilde{B}_2(f, d, i)$ in (S.14).

$$P(C) = \frac{1}{\sqrt{2\pi S^2}} \cdot \exp\left[-\frac{C^2}{2S^2}\right] \tag{S.19}$$

i.e.

$$S = S(N) = \frac{\gamma}{\sqrt{N}} \tag{S.20}$$

Note the direct relationship of the variance to the reciprocal of $\sqrt{N}$ as this plays a role below. We are interested in the PDF of the maximum correlation coefficient, $\hat{C}$ rather than the PDF for C itself. Based on the Gaussian above we find that the PDF for $\hat{C}$ is given by the following: [4, 7]

$$\phi\left(\hat{C}\right) = N_a P\left(\hat{C}\right) \left[\frac{1}{2}\left(1 + \operatorname{erf}\left(\frac{\hat{C}}{\sqrt{2S^2}}\right)\right)\right]^{N_a - 1}, \tag{S.21}$$

where N_a is the number of placement attempts when sliding one barcode across the other ($N_a = 2N$ for circular barcodes where the relative permutation and relative directions of the barcodes are not known) and $\operatorname{erf}(x)$ is the error-function. Note that for large N_a the expression above approaches the Gumbel distribution

presented in Sec. S.M.6 [8]. From equation (S.M.4.5) we can evaluate the expected maximum cross correlation ($m = 1$) and higher order moments ($m \geq 2$). We have:

$$\langle \hat{C}^m \rangle = \int_{-\infty}^{\infty} \hat{C}^m \rho \left(\hat{C} \right) d\hat{C} = \sqrt{\frac{1}{2\pi}} \left[S(N) \right]^m f\left(m, N_a \right) \tag{S.22}$$

where

$$f\left(m, N_a \right) = N_a \int_{-\infty}^{\infty} z^m \exp(-z^2/2) \left\{ \frac{1}{2} \left[1 + \text{erf} \left(\frac{z}{\sqrt{2}} \right) \right] \right\}^{N_a - 1} \tag{S.23}$$

For large N_a, the quantity $f(1, N_a)$ is well approximated by $c\sqrt{\ln(N_a)}$ with some constant c.[7]. Thus, using this result we obtain

$$\langle \hat{C} \rangle \propto \sqrt{\frac{\ln N_a}{N}} \tag{S.24}$$

We finally note that correlation coefficient values obtained by sliding one barcode across another are correlated over a few pixels [4]. Therefore, the quantities N_a and N above must be replaced by the effective sample sizes, $N_{\text{eff},a}$ and N_{eff} respectively. However, since these effective sample sizes are proportional to N[4], for large enough N, this does not change the maximum correlation coefficient's scaling with N.

S.M.4.6 Expected scaling of $\hat{C}$ for matching barcodes with respect to N

In the previous subsection, we found that the mean maximum correlation coefficient between "non-matching" barcodes is expected to decay with N. In this subsection, we address whether the maximum correlation coefficient when matching an experimental barcode to a theoretical barcode originating from the *same* DNA sequence exhibits any dependence on N.

Consider an experimental barcodes, $B_e\left(i\right)$, and a theoretical barcode, $B_t\left(i\right)$, for the same plasmid at different pixels $i = 0, \ldots, N - 1$. Neglecting bit-weights (i.e. assuming they are all 1), with some simple algebra where we subtract their respective means and divide them by their respective variances, we can rescale values for $B_e\left(i\right)$ and $B_t\left(i\right)$ to have means of 0 and variances of 1. Let us denote these rescaled barcodes as $\tilde{B}_e\left(i\right)$ and $\tilde{B}_t\left(i\right)$ and denote the element-wise difference of the rescaled barcodes by $\tilde{D}\left(i\right)$ such that we yield the following:

$$\tilde{B}_e\left(i\right) = \tilde{B}_t\left(i\right) + \tilde{D}\left(i\right) \tag{S.25}$$

Since the means are set to 0 and the variances are set to 1 for $\tilde{B}_e\left(i\right)$ and $\tilde{B}_t\left(i\right)$ we know the following to be true:

$$0 = \sum_i \tilde{B}_t\left(i\right) = \sum_i \tilde{B}_e\left(i\right) \tag{S.26}$$

$$N = \sum_i \tilde{B}_t\left(i\right)^2 = \sum_i \tilde{B}_e\left(i\right)^2 \tag{S.27}$$

Through equations (S.25) and (S.27), we yield the following:

$$N = \sum_i \tilde{B}_e(i)^2 = \sum_i (\tilde{B}_t(i) + \tilde{D}(i))^2 \tag{S.28}$$

Expanding the expression in equation (S.28) and substituting $\sum_i \tilde{B}_t(i)^2$ with N as seen in equation S.27, we find the following relation:

$$N = N + 2\sum_i (\tilde{B}_t(i)\,\tilde{D}(i)) + \sum_i (\tilde{D}(i)^2) \tag{S.29}$$

With a little algebraic manipulation on equation (S.29), we reach the following equality which serves as a direct consequence of having rescaled both barcodes:

$$\sum_i (\tilde{B}_t(i)\,\tilde{D}(i)) = -\frac{1}{2}\sum_i (\tilde{D}(i)^2) \tag{S.30}$$

Let us now consider the cross correlation coefficient, using equation (S.14), to study the difference of the maximum correlation coefficient values for a theoretical barcode against a theoretical barcode in comparison to a theoretical barcode against an experimental barcode that matches the theory. We have

$$\hat{C}_{tt} - \hat{C}_{et} = \frac{1}{N-1}\sum_i \tilde{B}_t(i)\,\tilde{B}_t(i) - \frac{1}{N-1}\sum_i \tilde{B}_e(i)\,\tilde{B}_t(i) \tag{S.31}$$

$$\hat{C}_{tt} - \hat{C}_{et} = \frac{1}{N-1}\sum_i \tilde{B}_t(i)\,(\tilde{B}_t(i) - \tilde{B}_e(i)) = -\frac{1}{N-1}\sum_i \tilde{B}_t(i)\,\tilde{D}(i) \tag{S.32}$$

$$\hat{C}_{tt} = \hat{C}_{et} + \frac{1}{2(N-1)}\sum_i \tilde{D}(i)^2 \tag{S.33}$$

We can see that the "advantage" that $\hat{C}_{tt}$ would have over $\hat{C}_{te}$ is almost exactly directly proportional to the mean of the squared differences between the barcodes (for large N). Based on this consideration, for long enough barcodes originating from the *same* DNA sequence, we do *not* expect significant length-dependence playing a part in the relationship between the maximum correlation coefficients produced by theoretical-experimental pairs ($\hat{C}_{et}$) and those produced by theoretical-theoretical pairs ($\hat{C}_{tt}$). Further, since $\hat{C}_{tt} = 1$ for two identical barcodes, we conclude that $\hat{C}_{et} \approx$ constant (N-independent) when comparing a theory barcode and an experimental barcode originating from the *same* DNA sequence. In the main text we find that this constant, for the current experimental setup, is $\hat{C}_{et} \approx 0.9$.

S.M.5 Consensus barcodes

As a result of stochasticity in individual experiments (for instance from the staining process, from thermal motion, and from optical noise), time-averages of kymographs and the barcodes directly produced from them are always expected to differ slightly from one another. Since molecule-to-molecule fluctuations are undesirable in characterizing plasmids from experiments, we here present a method for aligning and averaging data from the barcodes of multiple molecules that represent the same DNA sequence into "consensus barcodes." Our method also provides a means to identify out-of-class barcodes such that they may be prevented from erroneously affecting the consensus.

S.M.5.1 Scaling to a common length

When comparing a set of barcodes originating from M molecules with (presumably) identical DNA sequences, the fact that small variations in the lengths of the barcodes may still exist needs to be addressed. These small variations may arise for a number of reasons, including the possibility that the nanochannels have slightly differing diameters, resulting in one molecule being slightly more elongated than another. Our procedure for handling this is simply to stretch/compress all barcodes to a common target length equal to the mean length in pixels for the set of barcodes, see Sec. S.M.3. In subsequent subsections, we assume that all barcodes and bit-weights have been scaled to such a common target length. The number of pixels in these barcodes is denoted by N.

S.M.5.2 Overview of consensus barcode generation

After scaling the barcodes to a common length, we have M circular barcodes and their M associated bit-weighting barcodes, which we denote herein as $B_k\,(i)$ and $W_k\,(i)$ for $(k = 1, \ldots, M)$, respectively.

Here we describe an overview of our method for generating a consensus barcode from this data through agglomerative hierarchical clustering. First, we consider a collective pool of barcodes containing all of the molecular barcodes that we wish to merge into a consensus barcode. We then identify the two most similar barcodes in that pool of barcodes and merge them by replacing them by a weighted average of their values. This leaves us with a pool of $M - 1$ barcodes and we can repeat this agglomerative procedure until there is only a single barcode formed as a consensus from the pool of barcodes. For each iteration of this procedure, we keep track of which pairings were merged as well as the degree of similarity that was found for the merged pair. If the degree of similarity for a pair is considered too low, we may consider the two to represent different classes of barcodes. The "consensus barcode" for a class of barcodes is simply the barcode produced by merging all the in-class barcodes and none of the out-of-class barcodes using the agglomerative procedure described above.

We explore our implementation of the above procedure in greater mathematical detail in Secs. S.M.5.4 & S.M.5.5.

S.M.5.3 Consensus generation example

Here we illustrate our consensus method with an example using five ($M = 5$) barcodes, $B_1 - B_5$ from five different experiments on R100 plasmids where four of the barcodes are quite similar and one is not. Our procedure for generating a consensus produces data that can be structured as a binary tree of nodes containing barcodes as illustrated in Figure S9. We begin with "leaves" of the tree and use an agglomerative merging process to generate "parent" nodes in the tree which each contain the mergers of the two barcodes from their "child" nodes. Let it be noted that when we call nodes in the tree "parent" nodes or "ancestor" nodes we do not mean to imply any precedence within the generation process; on the contrary, these are generated from their descendant nodes since the process is agglomerative.

The order in which barcodes are chosen to be merged can effect the result. We merge the two most similar yet-to-be-merged barcodes together in each it-

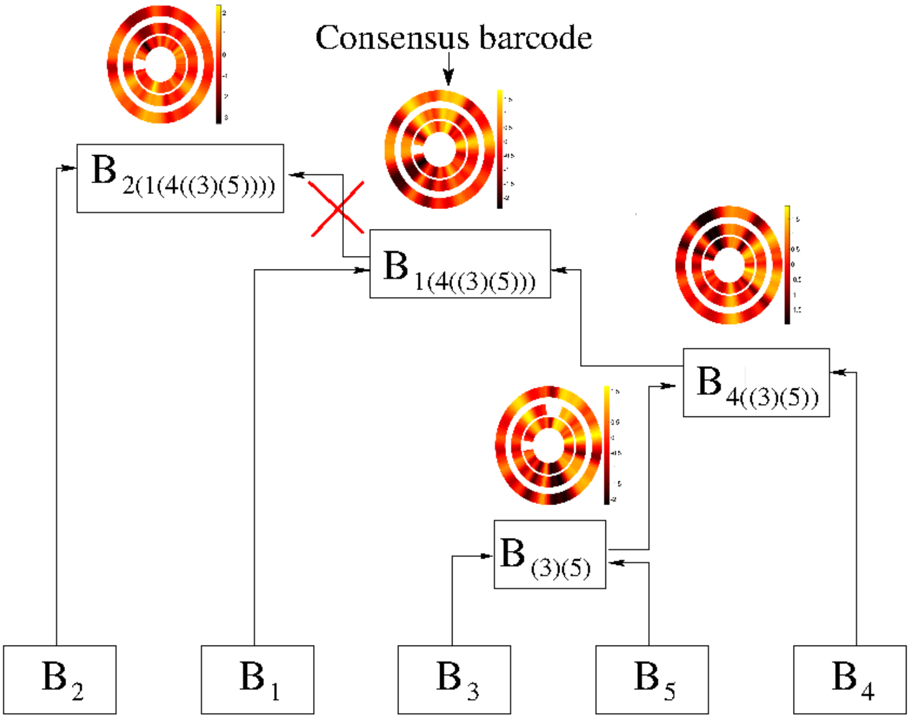

Figure S9: **Schematic illustration of our method for reducing molecule-to-molecule fluctuations using a hierarchical clustering type approach for consensus barcode generation**. (Top) Automated generation of consensus barcodes from five barcodes, B_1-B_5. Data are from experiments on the plasmid R100. The barcodes are successively merged pairwise, by finding the weighted average of each pair, until only the consensus barcode is left. The end-regions of the "best" pair is indicated as white regions in the circular plots. The two inner circles in these plots are the original barcodes, and the outer one is the merged barcode. The cut position and relative orientation of a barcode with respect to another one is determined by maximizing the score function, equation (S.34). Notice that full procedure for generating the consensus barcode, illustrated above, generates a binary tree structure, see Figure S11. By analyzing this binary tree (see Sec. S.M.5.6), and introducing a suitable cut-off value for the maximum correlation coefficient during the merging of two barcodes, the barcode B_2 is here deemed to be out of class, and, therefore, the consensus barcode is $B_{1(4((3)(5)))}$ in this example.

eration of our method, eventually building up to the root of the tree. The similarity score is computed for each possible pairing of barcodes (denoted as B_a and B_b) and is measured with a score, $S_{ab}\left(\hat{f}, \hat{d}\right)$ [equation (S.35)] which is elaborated on in Sec. S.M.5.4. The score involves finding the best alignment represented by the pair of values for $\hat{f}$ and $\hat{d}$ through the computation of correlation coefficient values for the bit-weighted barcodes at all potential alignments. The values computed for $\hat{f}$ and $\hat{d}$ for each pair of barcodes are used to circularly

shift and flip the barcodes as necessary to optimally align them. Post-alignment barcodes are shown as the two inner barcode rings at each parent node in Figure S9.

The outer ring at each parent node in Figure S9 represents the barcode produced by merging the barcodes. The process of merging barcodes is detailed in S.M.5.5 and involves taking an average of aligned barcode values. Our formulation is designed to give the values in the initial barcodes equal weight as any other barcode in the generated barcodes at each of their ancestor nodes. White regions in the barcode rings denote areas of the barcode where the bit-weight is 0. The value of the barcodes in these areas is ignored when generating a merged barcode.

In our example, we can see that B_3 and B_5 were the first to be merged together into $B_{(3)(5)}$. This indicates that they were considered the most similar barcodes from our initial set of barcodes. After that, the similarity of the newly generated barcode with B_4 was found to be greater than that of any other yet-to-be-merged barcodes, and they were merged to produce $B_{4((3)(5))}$. Next that new barcode was merged with B_1 to produce the consensus barcode in $B_{1(4((3)(5)))}$. The only other yet-to-be-merged barcode left was B_2. However, by comparing the two inner rings inside the result for $B_{2((4((3)(5))))}$, we see that B_2 was not very similar to the consensus barcode produced by others and was therefore excluded from the consensus. If the similarity score of a pair of barcodes fails to reach some threshold, we can choose to form consensuses below the root of the tree as we have done in this example. This idea is elaborated upon in Sec. S.M.5.6 where we discuss designating barcodes as “out-of-class"".

The resulting consensus barcode from all the barcodes other than the out-of-class barcode (B_2) is shown in greater detail in Figure S10. Instead of just showing the two immediate child barcodes, the four component barcodes (associated with the input barcodes at the descendant leaf nodes) are shown in their optimally aligned state.

S.M.5.4 Scoring barcode alignments

For each pair of barcodes from the pool, designated by a and b, when measuring similarity, all the possible alignments that can be produced by circularly shifting and flipping the barcodes relative to one another are explored to find the best possible alignment. The quality of an alignment for a pair of barcodes (B_a and B_b) with weights (W_a and W_b) is measured by the alignment score, $S_{ab}\left(f, d\right)$ [equation (S.34)]. Note that the Pearson correlation coefficient for barcodes a and b with alignment given by f and d, denoted here as $C_{ab}\left(f, d\right)$, is determined with the bit-weights associated with the barcodes taken into account such that the sample size, $n\left(f, d\right)$ [equation (S.8)] can vary depending on f and d if some bit-weights for a and b are 0. To help counterbalance the length dependency, we applied the square root correction factor seen in our equation. We apply this correction because we will be interested in the maximum score that can be produced by varying f and d and want the scores to be less dependent on the sample size. From our general analysis of the expected maximum correlation coefficient's dependency on sample size in Sec. S.M.4.5, neglecting the weaker logarithmic dependency on sample size, this square root correction is supported

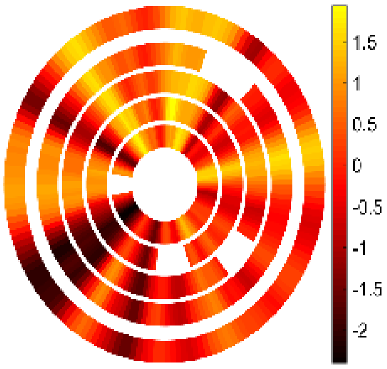

Figure S10: **Consensus barcodes are averages over optimally rotated and flipped experimental barcodes**. Four out of five barcodes (barcode 2 was excluded) from R100 plasmid experiments along with the resulting consensus barcode (outer cylinder) obtained. The end regions for the original barcodes are bit-weighted to zero and are displayed in white.

by equation S.24. Thus, our alignment score is defined as

$$S_{ab}(f,d) = \sqrt{n_{ab}(f,d)}C_{ab}(f,d)\,. \tag{S.34}$$

We denote the values for alignment parameters f and d that produce the maximum $S_{ab}(f,d)$ for a constant pair of barcodes a and b as $\hat{f}$ and $\hat{d}$. The associated alignment score is

$$S_{ab}\left(\hat{f},\hat{d}\right) = \max\{S_{ab}(f,d)\} \tag{S.35}$$

S.M.5.5 Merging barcodes and weights

With $\hat{f}$, $\hat{d}$ depending on the barcodes and weights for a and b, the score $S_{ab}\left(\hat{f},\hat{d}\right)$ tells us how similar the barcodes are when they are aligned. The pair of barcodes a and b which produces the maximum value for this score are regarded as the most similar and are therefore chosen to be merged. For the process of merging these circular barcodes, the values of $\hat{f}$ and $\hat{d}$ can be interpreted as instructions for how to manipulate the barcodes' orientations in order to synchronize their values to the best alignment. The pre-alignment index of B_a associated with an index i in the post-alignment version of B_a is denoted by $I_a(i)$ [equation (S.36)] and is dependent on a circular shift of $\hat{d}$. The pre-alignment index of B_b associated with an index i in the post-alignment version of B_b is denoted by $I_b(i)$ [equation (S.37)] and is dependent on $\hat{f}$ which specifies if flipping the barcode is necessary.

$$I_a(i) = \left(i - \hat{d}\right) \pmod N \tag{S.36}$$

$$I_b(i) = \begin{cases} i & \text{if } \hat{f} = 1 \\ \\ N - i & \text{if } \hat{f} = -1 \end{cases} \tag{S.37}$$

Once aligned, values for a pair of barcodes B_a and B_b with weights can be merged into a new barcode B_{ab} [equation (S.40)] by taking a weighted average. For the initial pool of barcodes, the weights (which we denote as $W_a^\star$ and $W_b^\star$ in this process) are the same as the initial bit-weights for the barcodes, W_a and W_b. For barcodes produced by merging a pair of barcodes, the weights denoted with asterisks are no longer restricted to just 0 and 1, but are instead the sums of the aligned weights for the pair of barcodes [equation (S.38)]. Note that the weights supplied to the similarity measure are however still bit-weights as presented in equation (S.39).

$$W_{ab}^\star(i) = W_a^\star(I_a(i)) + W_b^\star(I_b(i)) \tag{S.38}$$

$$W_{ab}(i) = \begin{cases} 1 & \text{if } W_{ab}^\star(i) > 0 \\ \\ 0 & \text{if } W_{ab}^\star(i) = 0 \end{cases} \tag{S.39}$$

$$B_{ab}(i) = \frac{W_a^\star(I_a(i))\, B_a(I_a(i)) + W_b^\star(I_b(i))\, B_b(I_b(i))}{W_{ab}^\star(i)} \tag{S.40}$$

It is important to note that with this weighted approach, if a consensus barcode is produced from a subset of barcodes from the initial pool, the order in which barcodes are merged does not effect the contribution of their respective values to the consensus barcode so long as all the barcodes are aligned in the same way; the consensus barcode is ultimately equivalent to an average of the values with non-zero bit-weights for the barcodes at each aligned index. Note also that the bit-weight associated with an index i in a barcode produced from multiple barcodes of the initial pool is 1 if any of the bit-weights at the aligned index for those barcodes is non-zero. This means that in practice, with reasonably bit-weighted data and a consensus of multiple barcodes, it is quite likely to have only 1s in the bit-weights for the consensus barcode.

S.M.5.6 Out-of-class barcode detection

As mentioned in the overview in Sec. S.M.5.2, the process of selecting the most similar barcodes, aligning them, and merging them can be repeated until all barcodes are merged together. Alternatively we can choose to terminate the process when all of the scores $S_{ab}\left(\hat{f}, \hat{d}\right)$ for all pairs of barcodes in the pool are below some threshold. The barcodes in the pool can then be considered to represent consensus for separate classes of barcodes.

Let us observe the binary tree in Figure S9 and introduce our method for determining ”bad” (out-of-class) barcodes. Such “bad” barcodes may originate from photo-cutting, failures in the staining process, or, simply because of “foreign” DNA molecules entering the sample. Note, however, that in the present study our workflow (see main text) is constructed such that we know that all molecules we study are intact ones. Hence, the molecule length is, most often, a very good classifier for detecting “foreign” molecules.

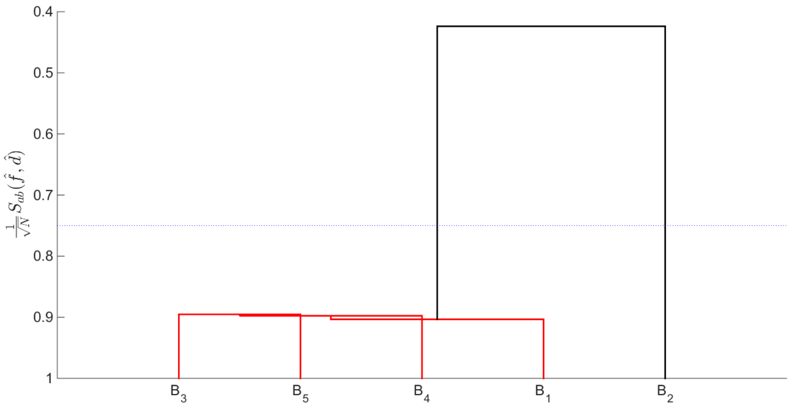

Figure S11: **Analysis of the binary tree generated through our consensus generation method allow us to detect out-of-class barcodes.** Binary tree generated from five R100 experimental barcodes (compare to Figure S9). The horizontal line corresponds a particular choice of threshold for classification purposes – different categories have different colors. The barcodes have been reshuffled to be adjacent to those in the same class. Note that B_2 is deemed to be out-of-class since it is not sufficiently similar to the consensus formed by the barcodes.

For binary tree analysis we here use standard methods from hierarchical clustering.[5, 6] If we add the similarity score values to the tree in Figure S9 at each merging point, we end up with a tree (dendrogram) as the one found in Figure S11. The vertical axis shows the alignment score scaled with respect to the sample length. The process of classifying barcodes now simply corresponds to "cutting the tree", i.e. choosing a threshold value for the maximum correlation coefficient – if a barcode pair has a smaller maximum correlation coefficient than the threshold they are deemed to be different and classified into separate categories. For our case, four out of the five R100 experimental barcodes are deemed to belong to the same category for a threshold value $S_{\text{threshold}} = 0.75\frac{1}{\sqrt{N}}$ (for plotting purposes we scale $S_{\text{threshold}}$ by the factor $1/\sqrt{N}$, where, here, $N = 168$). Barcode 2 is here classified as an out-of-class barcode – this particular barcode is also visually different from the rest (inner two rings at the top left cylindrical plot in Figure S9).

In the main text, we set $S_{\text{threshold}} = 0$ such that no cut is made and all plasmid barcodes of similar length are averaged into the consensus barcode.

Our approach to binary tree analysis for detecting out-of-class barcodes can be trivially adapted for the purpose of analyzing mixed samples containing different types of DNA molecules. If there are several independent subtrees below a threshold, barcodes may be classified as belonging to the same or different DNA molecule species as one another by noting whether their nodes belong to the same subtree. We plan to investigate the effectiveness of binary tree analysis for the purpose of classifying experimental samples in future studies.

S.M.6 Identifiability of plasmids based on the maximum correlation coefficient

Based on the similarity score, $\hat{C}$, introduced in previous sections, how can we quantify the quality of a "match" of a (individual, consensus or theory) barcode with respect to another barcode? To be able to answer this question, let us assume we have the following scenario: we matched an experimental, or theoretical, barcode to a set of K theoretical barcodes. This provides us with a set of K maximum correlation coefficient values, $\hat{C}_k$, $k = 1, \ldots, K$. Can we in such a scenario, define the number of "matches" in the K barcodes? Before answering this question, we first note that in [4] we introduced a p-value measure for answering a similar question, namely "is our maximum correlation coefficient value significantly larger than what you would expect by matching to random DNA sequence barcodes?". However, the p-value is subject to the usual "flaw" known to bioinformaticians [9], namely, that random sequences may not be very "representative" of real DNA sequences. In the present study we have a large reference database of theory barcodes. As we show below, this database allows us to circumvent some of the main potential problems of using random sequence barcodes as reference in the quest of attempting to identify "matches" between pairs of barcodes.

S.M.6.1 The Gumbel probability density function

Let us now introduce statistical means for defining the number of "identifiable" barcodes. For large enough K, the maximum correlation coefficients are expected to follow the Gumbel PDF (probability density for the maximum values) [9]

$$\phi(\hat{C}) = \frac{1}{\beta} \exp[-(y + e^{-y})] \tag{S.41}$$

where $y = y(\hat{C}) = (\hat{C} - \kappa)/\beta$, with parameters κ and β. Equation (S.41) follows from equation (S.M.4.5) in the large N limit. The mean of equation (S.41) is $\langle \hat{C} \rangle = \kappa + \beta\gamma$ where $\gamma \approx 0.5772$ is the Euler-Mascheroni constant. The variance of equation (S.41) is $\langle(\hat{C} - \langle \hat{C} \rangle)^2\rangle = \pi^2\beta^2/6$.

S.M.6.2 Parameter estimation

To be able to fit the parameters, κ and β, in equation (S.41) using the maximum cross correlation data, $\hat{C}_k$ ($k = 1, \ldots, K$), we proceed as follows (moment matching): First, we estimate β by equating the sample variance to the expression for $\langle(\hat{C} - \langle \hat{C} \rangle)^2\rangle$ given in Sec. S.M.6.1. The parameter κ is then obtained by equating the sample mean to the expression for the mean above. This procedure yields the following explicit estimate for the parameter β:

$$\beta = \frac{\sqrt{6}}{\pi}\sigma \tag{S.42}$$

where $\sigma^2 = [1/(\tilde{K} - 1)] \sum_{k=1}^{\tilde{K}} (\hat{C}_k - \mu)^2$, i.e., σ^2 is the sample variance of the $\hat{C}_k$s with mean $\mu = (1/\tilde{K}) \sum_{k=1}^{\tilde{K}} \hat{C}_k$. The parameter κ is subsequently determined by

$$\kappa = \mu - \beta\gamma \tag{S.43}$$

The quantity $\tilde{K}$ is estimated using two different approaches

1. *Non-recursive approach.* In this approach the sample mean and sample variances are estimated using the full data set, i.e. we set $\tilde{K} = K$ and use all $\hat{C}_k$ for $k = 1, \ldots, K$ when estimating the sample estimates for the mean and variance.

2. *Recursive approach.* In this method we first determine the sample mean and sample variances using the full data set (as in 1.). The criteria given below, equation (S.44), for determining the outliers in the data set are then applied, and the sample estimates recalculated with a reduced data set with no outliers included (i.e. with $\tilde{K} < K$ highest correlation coefficient values). This procedure is repeated until no additional outliers are detected.

Representative histograms alongside fitted $\phi(\hat{C})$ for a few different plasmids are displayed in Figure S12 (theory vs theory). Both the recursive as well as the non-recursive approach are used. Note that, by construction, the peak of the probability density is always located at the same or at a smaller cross correlation value for the recursive method as compared to the non-recursive approach.

Figure S12: **A plasmid is deemed an outlier if its cross correlation ends up in the tail of a fitted Gumbel PDF.** Illustration of the normalized histograms $\phi(\hat{C})$ and fitted Gumbel probability densities for (top, left) RP1, (top, right) R100, and (bottom,left) PUUH239.2. Both non-recursively (dashed curves) and recursively (solid curves) fitted curves are shown. Note that in the main text we exclusively use non-recursive fitting. (Bottom, right) Number of iterations in the recursive Gumbel parameter fitting procedure. Whenever this number = 1, the recursive and non-recursive approaches provides identical parameter estimates for the Gumbel parameters, β and κ.

S.M.6.3 Separability score

Based on the considerations in the previous two subsection, we now introduce our separability score for detecting outliers in the data set. Our definition is based on the fact that a maximum correlation coefficient which ends up "far out" in the tail of the fitted Gumbel distribution is deemed an outlier. To make this precise we say that we have an outlier if

$$\text{separability score}(\hat{C}) < \alpha \tag{S.44}$$

where α is a significance level. Our separability score is defined according to[1] separability score$(\hat{C}) = \int_{\hat{C}}^{\infty} \phi(\hat{C}')d\hat{C}'$, where $\phi(\hat{C})$ is the Gumbel probability density defined in equation (S.41). Using the known cumulative distribution for the Gumbel probability density we have the explicit expression:

$$\text{separability score}(\hat{C}) = 1 - \exp[-e^{-(\hat{C}-\kappa)/\beta}]. \tag{S.45}$$

[1]If $\phi(\hat{C})$ had been obtained using a set of random barcodes as reference, our separability score becomes a p-value [9].

Note that for the recursive method (2. above) the number of iterations in the method is dependent on α. Therefore, the separability score calculated using this method will, in general, be α dependent. In contrast, for the non-recursive method (1. above) the separability scores are always independent on α. In the main text we use the non-recursive method throughout.

References

[1] C. Noble, A.N. Nilsson, C. Freitag, J.P. Beech, J.O. Tegenfeldt and T. Ambjörnsson, "A Fast and Scalable Kymograph Alignment Algorithm for Nanochannel-Based Optical DNA Mappings", PloS One, vol. 10, e0121905 (2015).

[2] V.B. Teif and K. Rippe, "Calculating transcription factor binding maps for chromatin", Briefings in Bioinformatics, vol. 13, 187 (2011).

[3] V.B. Teif, "General transfer matrix formalism to calculate DNA–protein–drug binding in gene regulation: application to OR operator of phage λ", Nucleic acids research, vol. 35, e80 (2007).

[4] A.N. Nilsson, G. Emilsson, L.K. Nyberg, C. Noble, L. Svensson Stadler, J. Fritzsche, E.R.B. Moore, J.O. Tegenfeldt, T. Ambjörnsson, and F. Westerlund. "Competitive binding-based optical DNA mapping for fast identification of bacteria-multi-ligand transfer matrix theory and experimental applications on Escherichia coli." Nucleic acids research, vol. 42, e118 (2014).

[5] C.D. Manning, P. Raghavan and H. Schütze, "Introduction to Information Retrieval", Cambridge University Press (2008), chap. 17.

[6] F. Murtagh and P. Contreras, "Algorithms for hierarchical clustering: an overview", Wiley Interdisciplinary Reviews: Data Mining and Knowledge Discovery, vol. 2, 86 (2012).

[7] B. Schmittmann and R.K.P. Zia, "Weather records: Musings on cold days after a long hot Indian summer", American Journal of Physics, vol. 67, 1269 (1999).

[8] A.H-S. Ang and W.H. Tang, "Probability Concepts in Engineering Planning and Design, Vol. 2: Decision, Risk, and Reliability", John Wiley & Sons (1984).

[9] S. Karlin and S.F. Altschul, "Methods for assessing the statistical significance of molecular sequence features by using general scoring schemes", Proceedings of the National Academy of Sciences, vol. 87, 2264 (1990).
